# Cross-tissue omics analysis discovers ten adipose genes encoding secreted proteins in obesity-related non-alcoholic fatty liver disease

**DOI:** 10.1016/j.ebiom.2023.104620

**Published:** 2023-05-22

**Authors:** Nicholas Darci-Maher, Marcus Alvarez, Uma Thanigai Arasu, Ilakya Selvarajan, Seung Hyuk T. Lee, David Z. Pan, Zong Miao, Sankha Subhra Das, Dorota Kaminska, Tiit Örd, Jihane N. Benhammou, Martin Wabitsch, Joseph R. Pisegna, Ville Männistö, Kirsi H. Pietiläinen, Markku Laakso, Janet S. Sinsheimer, Minna U. Kaikkonen, Jussi Pihlajamäki, Päivi Pajukanta

**Affiliations:** a Department of Human Genetics, David Geffen School of Medicine at UCLA, Los Angeles, USA; b A. I. Virtanen Institute for Molecular Sciences, University of Eastern Finland, Kuopio, Finland; c Institute of Public Health and Clinical Nutrition, University of Eastern Finland, Kuopio, Finland; d Division of Cardiology, David Geffen School of Medicine at UCLA, Los Angeles, USA; e Vatche and Tamar Manoukian Division of Digestive Diseases, and Gastroenterology, Hepatology and Parenteral Nutrition, David Geffen School of Medicine at UCLA and VA Greater Los Angeles HCS, Los Angeles, USA; f Division of Pediatric Endocrinology and Diabetes, Department of Pediatrics and Adolescent Medicine, University of Ulm, Ulm, Germany; g Department of Medicine and Human Genetics, Division of Gastroenterology, Hepatology and Parenteral Nutrition, David Geffen School of Medicine at UCLA and VA Greater Los Angeles HCS, Los Angeles, USA; h Department of Medicine, University of Eastern Finland and Kuopio University Hospital, Kuopio, Finland; i Obesity Research Unit, Research Program for Clinical and Molecular Metabolism, Faculty of Medicine, University of Helsinki, Helsinki, Finland; j Obesity Center, Abdominal Center, Helsinki University Hospital and University of Helsinki, Helsinki, Finland; k Institute of Clinical Medicine, Kuopio University Hospital, University of Eastern Finland, Kuopio, Finland; l Department of Biostatistics, UCLA Fielding School of Public Health, Los Angeles, USA; m Department of Computational Medicine, David Geffen School of Medicine at UCLA, Los Angeles, USA; n Department of Medicine, Endocrinology and Clinical Nutrition, Kuopio University Hospital, Kuopio, Finland; o Bioinformatics Interdepartmental Program, UCLA, Los Angeles, USA; p Institute for Precision Health, David Geffen School of Medicine at UCLA, Los Angeles, USA

**Keywords:** Serum biomarkers, Non-alcoholic fatty liver disease, Obesity, Dual-tissue transcriptomics screening, Liver histology, Adipogenesis, *cis* regulatory variants

## Abstract

**Background:**

Non-alcoholic fatty liver disease (NAFLD) is a fast-growing, underdiagnosed, epidemic. We hypothesise that obesity-related inflammation compromises adipose tissue functions, preventing efficient fat storage, and thus driving ectopic fat accumulation into the liver.

**Methods:**

To identify adipose-based mechanisms and potential serum biomarker candidates (SBCs) for NAFLD, we utilise dual-tissue RNA-sequencing (RNA-seq) data in adipose tissue and liver, paired with histology-based NAFLD diagnosis, from the same individuals in a cohort of obese individuals. We first scan for genes that are differentially expressed (DE) for NAFLD in obese individuals’ subcutaneous adipose tissue but not in their liver; encode proteins secreted to serum; and show preferential adipose expression. Then the identified genes are filtered to key adipose-origin NAFLD genes by best subset analysis, knockdown experiments during human preadipocyte differentiation, recombinant protein treatment experiments in human liver HepG2 cells, and genetic analysis.

**Findings:**

We discover a set of genes, including 10 SBCs, that may modulate NAFLD pathogenesis by impacting adipose tissue function. Based on best subset analysis, we further follow-up on two SBCs *CCDC80* and *SOD3* by knockdown in human preadipocytes and subsequent differentiation experiments, which show that they modulate crucial adipogenesis genes, *LPL*, *SREBPF1*, and *LEP*. We also show that treatment of the liver HepG2 cells with the *CCDC80* and *SOD3* recombinant proteins impacts genes related to steatosis and lipid processing, including *PPARA*, *NFE2L2*, and *RNF128*. Finally, utilizing the adipose NAFLD DE gene *cis*-regulatory variants associated with serum triglycerides (TGs) in extensive genome-wide association studies (GWASs), we demonstrate a unidirectional effect of serum TGs on NAFLD with Mendelian Randomization (MR) analysis. We also demonstrate that a single SNP regulating one of the SBC genes, rs2845885, produces a significant MR result by itself. This supports the conclusion that genetically regulated adipose expression of the NAFLD DE genes may contribute to NAFLD through changes in serum TG levels.

**Interpretation:**

Our results from the dual-tissue transcriptomics screening improve the understanding of obesity-related NAFLD by providing a targeted set of 10 adipose tissue-active genes as new serum biomarker candidates for the currently grossly underdiagnosed fatty liver disease.

**Funding:**

The work was supported by 10.13039/100000002NIH grants R01HG010505 and R01DK132775. The Genotype-Tissue Expression (GTEx) Project was supported by the Common Fund of the 10.13039/100000179Office of the Director of the 10.13039/100000002National Institutes of Health, and by 10.13039/100000054NCI, 10.13039/100000051NHGRI, 10.13039/100000050NHLBI, 10.13039/100000026NIDA, 10.13039/100000025NIMH, and 10.13039/100000065NINDS. The KOBS study (J. P.) was supported by the 10.13039/501100013500Finnish Diabetes Research Foundation, 10.13039/501100004092Kuopio University Hospital Project grant (EVO/VTR grants 2005–2019), and the 10.13039/501100002341Academy of Finland grant (Contract no. 138006). This study was funded by the European Research Council under the European Union’s Horizon 2020 research and innovation program (Grant No. 802825 to M. U. K.). K. H. P. was funded by the 10.13039/501100002341Academy of Finland (grant numbers 272376, 266286, 314383, and 335443), the 10.13039/100008723Finnish Medical Foundation, Gyllenberg Foundation, 10.13039/501100009708Novo Nordisk Foundation (grant numbers NNF10OC1013354, NNF17OC0027232, and NNF20OC0060547), 10.13039/501100013500Finnish Diabetes Research Foundation, 10.13039/501100005633Finnish Foundation for Cardiovascular Research, 10.13039/100007797University of Helsinki, and Helsinki University Hospital and Government Research Funds. I. S. was funded by the 10.13039/501100008413Instrumentarium Science Foundation. Personal grants to U. T. A. were received from the Matti and Vappu Maukonen Foundation, Ella och Georg Ehrnrooths Stiftelse and the 10.13039/501100005633Finnish Foundation for Cardiovascular Research.

**Research in context**

**Evidence before this study**

Leading theories suggest that adipose tissue dysfunction plays a large role in obesity-related non-alcoholic fatty liver disease (NAFLD). Adipose and liver gene expression have separately been associated with NAFLD, and serum biomarker candidates (SBCs) for NAFLD have previously been proposed, though their accuracy is limited. However, not much is known about NAFLD genes that have adipose tissue specific activity and encode secreted proteins, which could potentially be traced in serum to noninvasively diagnose NAFLD. To assess the existing understanding of potential adipose NAFLD biomarker genes, we searched PubMed with the following terms: (adipose origin NAFLD) AND ((biomarker) OR (serum) OR (diagnostics) OR (primary care) OR (multi tissue omics) OR (multi tissue RNA-seq)), including results from all dates up to October 19, 2022. This search returned 21 articles. Of these 21 studies, none utilised RNA-seq data from multiple human tissues to study the effects of adipose tissue dysfunction on NAFLD.

**Added value of this study**

We leveraged a dual-tissue RNA-seq obesity cohort with adipose tissue and liver biopsy samples and liver histology available to discover a set of 10 adipose enriched NAFLD SBCs. To the best of our knowledge, our study is one of the first to leverage multi-tissue RNA-seq data in humans to study adipose origin NAFLD. We identified the SBCs using NAFLD differential expression analyses in adipose and liver tissue, followed by best subset analysis. We demonstrated that key SBCs may induce or suppress adipogenesis via longitudinal siRNA knockdown in human preadipocytes differentiated to adipocytes. We also show that adipose-origin NAFLD is linked to elevated serum TGs, which are influenced by *cis* variants regulating SBC expression in adipose tissue. Thus, our findings contribute significantly to the existing body of work on adipose-origin NAFLD by pinpointing individual genes whose NAFLD-associated expression can be traced specifically to the adipose tissue and potentially detected in serum to diagnose NAFLD early in its development.

**Implications of all the available evidence**

By discovering 10 SBC genes that may play a role in the onset of NAFLD triggered by adipose dysfunction, we improve the overall understanding of the NAFLD mechanisms in obese patients. Our genes also have the potential to be developed into a serum biomarker panel that could be used to diagnose NAFLD in a cheaper and less invasive method than is currently possible, overall improving patient health by avoiding adverse downstream outcomes, including fibrosis and cirrhosis. The feasibility of these biomarker genes will be further assessed by testing the association of their serum protein levels with NAFLD.

## Introduction

Non-alcoholic fatty liver disease (NAFLD) is a highly prevalent disorder that affects ∼25% of people globally.^1^ NAFLD represents a heterogeneous spectrum of liver disease, ranging from simple steatosis to liver fibrosis and non-alcoholic steatohepatitis (NASH).^2^ NAFLD can ultimately lead to liver cirrhosis, and is expected to become the leading cause of liver transplantation within this decade.^3^ Heterogeneity in NAFLD etiology and pathogenesis is also reflected by the fact that while obesity is the key risk factor for NAFLD, 5%–40% of NAFLD patients are normal weight, depending on the population.^4^

NAFLD manifests in the liver, but prevailing theories suggest that the obesity-driven form of NAFLD originates in adipose tissue.^2^^,^^5^^,^^6^ It has been hypothesised that some obese individuals cannot generate new adipocytes effectively enough (hyperplasia) to store extra fat, and instead their existing adipocytes become larger (hypertrophy).^2^^,^^5^ These large adipocytes tend to undergo cellular death, attracting infiltrations of inflammatory cells, such as macrophages, which ultimately causes low-grade inflammation and deteriorates adipose tissue functions.^2^^,^^5^ As a result, adipose lipolysis increases, releasing free fatty acids into the bloodstream^2^^,^^5^^,^^7^ This drives ectopic fat deposits onto vital organs, including the liver. These deposits evoke macrophage infiltration and inflammation in the liver, which the liver attempts to repair with scar tissue, i.e. fibrosis.^2^^,^^5^ Without weight loss intervention, the obese adipose tissue becomes increasingly dysfunctional, and the liver becomes increasingly fibrotic, until the liver is permanently damaged.^2^^,^^5^ Existing evidence broadly supports this hypothesis,^2^^,^^6^^,^^7^ but many of the exact molecular factors driving NAFLD pathogenesis remain unknown. Furthermore, the known common NAFLD variants, including the *PNPLA3*, *TM6SF2*, *HSD17B13*, and *GCKR* variants, explain only a small proportion (10%–20%) of its heritability.^8^

Presently, there is no effective treatment for the obesity-driven advanced forms of NAFLD; however, as simple steatosis is still reversible through weight loss, early diagnosis would be critically important.^2^ Although a variety of diagnostic strategies currently exist for the various stages of the NAFLD spectrum, these strategies are either too invasive (e.g. liver biopsy) or too expensive (e.g. magnetic resonance imaging (MRI)) to be broadly implemented in primary health care.^2^^,^^9^ Liver biopsy can accurately identify the stages of steatosis, fibrosis, and NASH by direct histological assessment of the liver tissue.^2^ However, a liver biopsy is invasive, relatively risky, and prone to bias.^2^^,^^10^ Imaging methods, including abdominal ultrasonography, MRI, and elastography, are less invasive.^2^^,^^9^ However, MRIs are expensive, and ultrasonography and elastography have low sensitivity in detecting early steatosis cases and cannot robustly detect NASH.^2^ Existing serum biomarker panels^2^^,^^9^^,^^11^ are noninvasive and inexpensive. However, predictive power of these models remains limited, with area under the receiver operating curve ranging from 0.66 to 0.87.^2^^,^^9^ Because of these diagnostic challenges, the early stages of NAFLD go largely underdiagnosed, and many patients already exhibit fibrosis by the time NAFLD is detected.

To elucidate the role of adipose tissue gene expression in NAFLD pathogenesis and identify adipose-origin serum biomarker candidates (SBCs) for NAFLD, we used a cross-tissue omics approach that utilises dual-tissue transcriptomic data and liver histology from a cohort of individuals with morbid obesity who underwent bariatric surgery. To follow up our key cross-tissue transcriptomics findings, we performed functional analysis knocking down SBCs during preadipocyte differentiation (i.e. adipogenesis), treated human liver HepG2 cells with recombinant SBC proteins, and conducted a Mendelian randomization (MR) analysis for adipose-origin NAFLD in the large UK Biobank. We found that knockdown of the SBC Coiled-Coil Domain Containing 80 (*CCDC80*) significantly increased the expression of the fatty acid synthesis master transcription factor Sterol Regulatory Element Binding Transcription Factor 1 (*SREBF1*),^12^ and knockdown of the SBC Superoxide Dismutase 3 (*SOD3*) significantly decreased the expression of the satiety signalling protein Leptin (*LEP*).^13^ Additionally, we found that treatment of the HepG2 cells with the *CCDC80* recombinant protein significantly decreased the expression of the fatty acid metabolism transcription factor, *PPARA*,^14^^,^^15^ and treatment with the *SOD3* recombinant protein significantly decreased the expression of the steatosis-associated gene, *RNF128*.^16^ We also demonstrated a possible adipose-origin unidirectional effect of serum triglycerides (TGs) on NAFLD in MR. Our study has the potential to substantially improve patient outcomes by discovering genes which may contribute to the pathogenesis of obesity-induced, adipose-origin NAFLD, and could be developed into a serum biomarker panel to noninvasively detect NAFLD.

## Methods

### Study cohorts

#### Kuopio Obesity Surgery Study (KOBS) cohort used for Weighted Gene Co-expression Network Analysis (WGCNA), Differential Expression (DE) analysis, best subset analysis, and MR analysis

The KOBS cohort was recruited at the University of Eastern Finland and Kuopio University Hospital among Finnish individuals with morbid obesity who underwent bariatric surgery, as described in detail previously.^17^, ^18^, ^19^, ^20^ The mean age of the KOBS cohort is 49 years (±9 years), and comprises 78 males (30%) and 181 females (70%) (self-reported). The inclusion criteria were: body mass index (BMI) ≥ 40 kg/m^2^, or BMI ≥ 35 kg/m^2^ with an obesity-associated comorbidity. Individuals with surgical contraindications were excluded. During the surgery, subcutaneous adipose and liver biopsies were collected for bulk RNA-sequencing as well as serum samples for clinical measurements, as described in detail previously.^21^ Briefly, the KOBS participants have detailed phenotype data measured for liver histology, metabolic, and anthropometric traits. These include age, sex, BMI, serum lipid and glucose levels, and liver histology assessments related to NAFLD (i.e. liver steatosis grade, fibrosis stage, and NASH), diagnosed as described in detail previously.^17^, ^18^, ^19^, ^20^ KOBS genotype data were generated using an Illumina HumanOmniExpress BeadChip, as previously described.^21^^,^^22^ All individuals in the KOBS cohort provided a written informed consent, and the study protocols were approved by the local ethics committee.

#### UK Biobank (UKB) cohort used for MR analysis

The UKB cohort is a large cohort of individuals from the UK (n = 502,617), collected beginning in 2006. In this study, we used the set of unrelated individuals of European ancestry (n = 392,551; ∼54% self-reported female). We utilised the genotype data for these individuals, which were collected using two different genotype arrays spanning over 800,000 variants, as described earlier.^23^ The genotype data were quality controlled as described previously^11^ before being used for GWAS analysis.

#### METabolic Syndrome In Men (METSIM) cohort used as a linkage disequilibrium (LD) reference

The METSIM study was conducted at the Kuopio University Hospital and University of Eastern Finland, and enrolled Finnish males (self-reported) aged 45 to 73 (n = 10,197) as previously described.^21^^,^^24^ All participants provided written informed consent, and the study protocols were approved by the local ethics committee. In this study, we used previously collected genotype data from 6686 unrelated individuals, generated using an Illumina HumanOmniExpress BeadChip.^21^^,^^24^

We used these three existing cohorts without employing any sex-based criteria for our study design. We adjusted for sex as a covariate in the WGCNA, DE, and best subset analyses described below.

#### Genotype imputation and quality control in the KOBS and METSIM cohorts

We performed a series of quality control steps on the KOBS and METSIM genotype data using PLINK v1.9,^25^ as described previously with minor modifications.^21^^,^^22^ In short, we removed SNPs that were strand ambiguous, unmapped, monomorphic, had high missingness, failed the Hardy–Weinberg equilibrium test, or had low minor allele frequency. We also removed individuals with mismatches between reported and imputed chromosomal sex. Imputation was run on the Michigan Imputation Server, as described previously with minor modifications.^21^^,^^22^

#### Adipose and liver bulk RNA sequencing in the KOBS cohort

The adipose RNA-seq data^17^ (n = 262) were generated by sequencing TruSeq stranded libraries on the HiSeq4000 sequencing platform, producing an average of 42.38 M reads.^17^ The liver RNA-seq data^19^ (n = 267) were generated by sequencing Ribo-Zero stranded libraries on the HiSeq2500 sequencing platform, producing an average of 39.73 M reads.^19^ We aligned both the adipose and liver bulk reads to the GRCh37/hg19 reference using a 2-pass pipeline with STAR,^26^ quantified the mapped reads using the Subread v1.6.2 package featureCounts, and performed QC using PicardTools.^27^

#### Identification of adipose and liver cell-type marker genes

To identify cell-type marker genes in adipose tissue and liver, we leveraged two additional cohorts with existing single nucleus RNA sequencing (snRNA-seq) data.

In the subcutaneous adipose cohort, snRNA-seq was performed on subcutaneous adipose biopsies from 15 individuals in the Finnish Twin and CRYO studies, as described in detail previously.^22^ The 15 individuals had a mean age of 33 years (±7 years), and were comprised of 6 males (40%) and 9 females (60%) (self-reported). All individuals provided written informed consent, and the study protocols were approved by the local ethics committee. Filtering was performed with DIEM,^28^ and clusters were identified using Seurat v3.2.3.^29^ Cell-type annotation was performed using SingleR v1.2.4,^30^ and cell-type marker genes were selected based on a Wilcoxon rank-sum test.^22^

In the liver snRNA-seq cohort, female patients (n = 3, self-reported), with a mean age of 78 years (±3 years), underwent surgery at the Dumont-UCLA Liver Cancer Center to treat hepatocellular carcinoma (HCC), as described in detail previously.^31^^,^^32^ All participants provided written informed consent, and the study protocols were approved by the UCLA IRB. During the surgery, tumor and adjacent non-tumor biopsies were collected. In the present study, we used only the snRNA-seq samples from non-tumor tissue. To identify marker genes for each liver cell-type, we tested normalised expression between nuclei within and outside a cluster. We normalised raw counts by first scaling all nuclei to sum to 1,000, then log-transforming. Next, we used the FindAllMarkers function from Seurat^29^ to run differential expression. For each cell-type, we performed a logistic regression for each gene testing expression of nuclei within the cell-type against those classified as any other cell-type. We kept marker genes with an average log2 fold change of at least 0.1. We corrected p-values for multiple testing across all genes and cell-types using false discovery rate (FDR).

#### Existing TG and NAFLD GWAS summary level data in the UKB cohort used for the Mendelian randomization analyses

For our MR analysis, we leveraged our previously published GWAS summary statistics for TGs and NAFLD.^11^ Serum TGs have been measured in the UKB cohort, and for the NAFLD GWAS, we have generated an imputed NAFLD status, as described in detail previously.^11^ Briefly, imputed NAFLD scores (NAFLDS) were modelled for individuals in the UKB cohort using an elastic net regression, and the imputed NAFLD status was derived using cutoffs of NAFLDS.^11^ The NAFLDS score was validated with 100-fold cross-validation. GWAS was then performed on TGs and imputed NAFLD status, using a linear mixed model implemented by BOLT-LMM, as described previously.^11^

#### Identification of adipose and liver cis-eQTLs in the KOBS cohort

To identify genetic variants associated with gene expression in the KOBS adipose and liver data, we ran *cis*-eQTL analysis using the R package Matrix eQTL.^33^ To prepare the expression data, we first computed fragments per kilobase of transcript per million mapped reads (FPKMs) from the adipose and liver raw counts, which were quantified using featureCounts as described above. Next, we performed a rank-based inverse normal transformation on the FPKMs for each gene, and conducted probabilistic estimation of expression residuals (PEER) analysis while correcting for common RNA-seq technical factors. The adipose FPKMs were corrected for 25 PEER factors, while the liver FPKMs were corrected for 10 PEER factors, as described previously with minor modifications.^21^^,^^22^ A subsequent inverse normal transformation was performed on the PEER-corrected FPKMs.

To compute adipose and liver *cis*-eQTLs, we ran Matrix eQTL in linear mode on the KOBS imputed genotypes and corrected FPKMs, defining the *cis* regions as ±1 Mb from the end of each gene and otherwise using the default parameters. We defined significant *cis*-eQTLs in each tissue as those with FDR < 0.05.

#### WGCNA of KOBS adipose and liver expression data

To investigate molecular crosstalk between subcutaneous adipose tissue and liver, we used the KOBS expression data to construct weighted gene correlation networks with the R package WGCNA^34^ v1.70. In this and all other statistical analyses, we utilised the R package tidyverse,^35^ including ggplot2, extensively. Before creating the networks, we first normalised the expression data according to the developers’ instructions for RNA-seq data. Briefly, we selected genes with nonzero expression in 90% of samples (as described previously^36^), calculated their counts per million (CPM), performed an inverse normal transformation, regressed out common RNA-seq covariates (age, sex, RNA integrity number, percent uniquely mapped reads, percent intronic bases, and median 3’ bias), and thereafter performed a second inverse normal transformation. We used the 90% over zero filter because genes with a measured expression value of 0 in most samples typically display non-normal bimodal expression distributions, which cannot be fully corrected by inverse normal transformation. This resulted in 21,408 and 22,500 input genes in the adipose tissue and liver, respectively. A total of 17,523 genes were shared between the input genes in the adipose tissue and liver (82% of the adipose genes and 78% of the liver genes, respectively). After normalisation, we verified that no extreme outliers existed in the data by hierarchically clustering the samples.

Next, we constructed two independent co-expression networks, one in the subcutaneous adipose tissue and one in the liver, using WGCNA. We followed the “step-by-step network construction” tutorial from the WGCNA website, which involved calculating an adjacency matrix, converting it to a dissimilarity topological overlap matrix (TOM), clustering genes hierarchically based on the TOM, performing a dynamic tree cut, and merging modules based on their module eigengene correlation. When constructing the adjacency matrix, we used a soft threshold power of 7 and 10 in adipose and liver, respectively, based on inspection of the plots showing the effect of soft threshold power on mean connectivity and scale free topology model fit. When merging modules, we used a cut height of 0.10 and 0.25 for adipose and liver, respectively, based on inspection of the module eigengene dendrograms. The completed networks contained 57 and 28 modules for adipose and liver, respectively.

With the two networks constructed, we followed the “Relating modules to external clinical traits” tutorial to correlate all module eigengenes in both networks with relevant metabolic and histological phenotypes: liver steatosis, liver fibrosis, NASH diagnosis, type II diabetes (T2D), statin usage, BMI, TGs, and fasting glucose adjusted for T2D. We assessed the significance of these correlations after Bonferroni correction. Additionally, we correlated the module eigengenes of both networks with each other, and assessed the significance of these correlations after Bonferroni correction.

Finally, we calculated the functional enrichment of modules in both networks. First, we calculated the KEGG pathway enrichment using an overrepresentation analysis in WebGestalt^37^ 2019. Next, we calculated the enrichment (compared to all genes with nonzero expression in 90% of samples) of adipose aware DE genes; unique cell-type marker genes for adipocytes, preadipocytes, and hepatocytes; and genes which were both DE and unique cell-type markers, respectively, using a hypergeometric test. We defined adipose aware DE genes as genes DE in adipose tissue but not in the liver between the individuals with histology-based healthy liver and those with NAFLD. We also identified transcription factors in the modules using PANTHER^38^ v16. We calculated the module membership of key genes identified in the functional enrichment tests using a Pearson correlation with the module eigengene.

#### DE analysis of KOBS adipose and liver expression data

To identify genes DE between the KOBS participants with and without NAFLD, we performed case-control DE analysis on KOBS adipose and liver expression data for steatosis, fibrosis, and NASH, diagnosed by liver histology.^17^, ^18^, ^19^ In each analysis, the cases were patients with a nonzero grade for the liver histology phenotype being tested (n = 158, 118, and 85 for steatosis, fibrosis, and NASH, respectively). The controls were patients with a grade of zero in all three liver histology phenotypes (n = 87 for all tests).

To prepare for the DE analysis, we performed trimmed mean of M values (TMM) normalization on the adipose and liver bulk RNA-seq data using edgeR^39^ v3.32.1. To run the DE analysis, we then input these normalised expression values into the limma-voom pipeline^40^ v3.46.0, correcting for the same covariates that were regressed out in the WGCNA analysis. We assessed the significance of DE genes using FDR < 0.05. After identifying the DE genes for steatosis, fibrosis, and NASH in adipose and liver tissue, we calculated the enrichment of cell-type marker genes in all DE gene lists using a hypergeometric test.

#### Filtering of adipose NAFLD DE genes for adipose-origin serum biomarker candidates

To identify adipose-origin SBCs for NAFLD, we applied a filtering approach that focused on the adipose NAFLD DE genes. We started with the list of genes which were DE for any of the three liver histology traits (steatosis, fibrosis, or NASH) in the subcutaneous adipose tissue. Next, we removed the genes that were also DE for any of the same NAFLD traits in the liver. We then downloaded tissue-specific median transcripts per million (TPM) data from GTEx, and selected the genes that had both the median TPM > 30 in subcutaneous adipose tissue and whose ratio of subcutaneous adipose median TPM to liver median TPM was >10. Finally, we selected the genes that encoded proteins secreted to serum, based on the HPA list of secreted proteins.^41^ We designated the adipose NAFLD DE genes that satisfied all of these filters as SBCs.

To assess the relationship of the SBCs to each other, we correlated their adipose expression. First, we normalised the data by calculating the log-CPM of all SBCs. Then, we computed the Pearson correlation of every pairwise combination of SBCs using the R package Hmisc^42^ v4.6, and assessed the significance of each correlation after Bonferroni correction.

#### Best subset approach to identify key SBCs

To find the most effective subset of SBCs, we tested the proportion of variance in steatosis, fibrosis, and NASH explained by the adipose expression of different combinations of SBCs, using the *leaps* algorithm. To normalise the data, we first calculated the adipose CPM of the SBCs, and then performed an inverse normal transformation. Next, we fit linear models in a best subset analysis to test the variance in NAFLD traits explained by adipose SBC expression, employing the regsubsets function from the R package leaps^43^ v3.1. This package is implemented with an iterative algorithm which identifies the best-fitting linear model with each number of genes included, ranging from a single variable to every variable provided.

For fibrosis and NASH, we tested all SBC genes DE for the target phenotype as possible inputs to the model. For steatosis, to identify genes involved in the early onset of NAFLD, we only tested SBC genes exclusively DE for steatosis, and not fibrosis or NASH. We included RNA-seq covariates (the same used in WGCNA and DE) in these analyses by regressing them out of the transformed CPMs before running leaps.

We identified the models that explained maximum variance in steatosis, fibrosis, and NASH using the Bayesian Information Criterion (BIC), and assessed the significance of these models with a permutation test (number of permuted sample sets, B = 100,000). For each permutation, we selected a random set of adipose genes with nonzero expression in 90% of samples, equal to the number of genes in the best subset model chosen by leaps. We then used a linear model to test the variance in the phenotype being assessed that was explained by the adipose expression of those genes. The p-value for each SBC model was defined as the proportion of random permuted models whose r^2^ value was greater than the SBC model.

#### Simone Golabi Behmel Syndrome (SGBS) cell culture

The SGBS human preadipocyte cells were obtained from Dr. Martin Wabitsch, University of Ulm, Ulm, Germany, who validated these cells, as described previously.^44^ No evidence of any mycoplasma contamination was observed during the culture or differentiation period. SGBS preadipocyte cells^44^ were maintained in DMEM/F-12 Nut media (Lonza # BE12-719F) with 4 μg/ml Pantothenate (Sigma, #P-5155), 8 μg/ml Biotin (Sigma #B-4639), 10% fetal bovine serum (FBS), 1% penicillin-streptomycin. These cells undergo complete differentiation into mature adipocytes in 14 days.^45^ When pre-adipocytes reached confluence they were treated with serum free differentiation medium DMEM/F-12 supplemented with 4 μg/ml Pantothenate, 8 μg/ml Biotin, 1% penicillin-streptomycin, 2 μmol/l rosiglitazone (Cayman Chemical # CAT 71740), 25 nmol/l dexamethasone (Sigma # D-4902), 0.5 mmol/l methylisobuthylxantine (Sigma #I5879), 0.1 μmol/l cortisol (Sigma #H0888), 0.01 mg/ml transferrin (Sigma #T8158), 0.2 nmol/l triiodotyronin (Sigma #T6397), and 20 nmol/l human insulin (Sigma #I9278) for 7 days. This was followed with cell culture in adipogenic medium DMEM/F-12 supplemented with 4 μg/ml Pantothenate, 8 μg/ml Biotin, 1% penicillin-streptomycin, 0.1 μmol/l cortisol, 0.01 mg/ml transferrin, 0.2 nmol/l triiodothyronine, and 20 nmol/l human insulin for an additional 7 days.

#### CCDC80 and SOD3 siRNA knockdown and sample collection for RNA-seq experiment

The cells were seeded in a 6-well plate at 1.6 × 106 cells per well. Once the cells reached 50% confluency, they were transfected with siRNA using lipofectamine RNAiMAX (Invitrogen) according to the manufacturer’s instructions. Predesigned siRNAs from Thermo Fisher Scientific were used [scrambled (control) siRNA (30 nM) (ref no: 4390843), *CCDC80* (60 nM) (ID: s45625), *SOD3* (60 nM) (ID: s13272)].

During differentiation, the cells were devoid of serum and thus they stop dividing. This enables the cells to retain the siRNA transfection mix for up to 14 days, as previously shown.^46^ In this study the cells were differentiated, and the samples were collected at different timepoints. The cells were incubated with the transfection mix for 48 h, after which the baseline samples were collected. The rest of the samples were treated with differentiation media (as described above), and collection was done at 24 h, 4 days, and 7 days.

#### Oil Red O staining of siRNA knockdown experiment samples

The cells were seeded in a 12-well plate at 8 × 10^4^ cells per well. Silencing of *CCDC80* and *SOD3* was done as described above, and samples were collected at the baseline, 24 h, 4 days, and 7 days, and stained with Oil Red O (ORO). The cells were washed with 1 × DPBS twice and fixed with 4% paraformaldehyde for 30 min at room temperature (RT). The cells were rinsed 2x with distilled H_2_O, followed by incubation for 5 min in 60% isopropanol. The cellular neutral lipids were stained with ORO (0.5% ORO in 100% isopropanol) for 20 min at RT. The cells were rinsed, then counter stained with hematoxylin for 1 min, and excess stain was washed off. The cells were visualised using the EVOS Core XL microscope. The ORO stain from the preadipocytes and adipocytes was extracted using 100% isopropanol. The staining intensity of ORO was measured at 492 nm and normalised to the cell number.

#### siRNA knockdown RNA-seq library preparation

Cells were lysed and RNA was extracted using miRNeasy micro kit (Qiagen). Library samples were prepared using QuantSeq 3’ mRNA-seq library prep kit FWD (Lexogen) according to the manufacturer’s instructions, amplified for 18 cycles, and then sequenced with Illumina Next seq 500 for 75 cycles.

#### Alignment and quantification of siRNA knockdown RNA-seq data

We aligned raw QuantSeq RNA-seq reads from the siRNA knockdown experiment to the GENCODE GRCh37 human reference genome and annotation v19 using STAR v2.5.2. We measured control, scrambled (control) siRNA, *CCDC80* siRNA knockdown, and *SOD3* siRNA knockdown conditions across the four differentiation time points, with 3–4 replicates per condition, resulting in a total of 59 samples. Before running the alignment, we first trimmed the raw reads with cutadapt v3.5, using a polyA sequence concatenated to the standard Illumina adapter as the trimming target. We used a 2-pass method to align the trimmed reads, which had an average read length of 83.5bp. After alignment, we verified the quality of our data using FastQC, based on statistics including sequence quality, GC content, and adapter content. Finally, we quantified gene expression using the Subread v1.6.2 package featureCounts, and selected only uniquely mapped reads for the expression data.

#### DE analysis of siRNA knockdown expression data

To identify genes DE between the *CCDC80* and *SOD3* knockdown samples and control samples, we performed DE analysis of the knockdown experiment expression data. First, we removed lowly expressed genes by selecting only those which had a total count of >10 summed across the samples within one group (control or knockdown). Next, we restricted the genes being tested for DE to *SOD3*, *CCDC80*, unique cell-type marker genes for adipocytes and preadipocytes, and adipogenesis pathway genes downloaded from WikiPathways^47^ WP236 (n = 492 genes tested). We also excluded the non-transfected control samples, resulting in a final sample size of 28 for both *CCDC80* and *SOD3*.

We ran the limma-voom pipeline on the knockdown expression data in the same way as described for the KOBS DE analyses, except without including any covariates, thus comparing the knockdown samples to the scrambled (control) siRNA samples independently at each time point and for each knockdown condition (n = 7 in all 8 tests). Our rationale for not including typical technical covariates of RNA-seq is that this was an *in vitro* cell-line experiment with isogenic replicates, in which the expression was assessed by performing QuantSeq 3’ tag-based sequencing instead of the regular, highly dynamic bulk RNA-seq analysis.

To interpret the results of the DE tests, we analysed the lists of DE genes (n = 492 tested genes) at each time point. First, we verified that the knockdown was successful using the p-value of p < 0.05 for *CCDC80* and *SOD3* in all timepoints from the corresponding experiments. Then we assessed the significance of the DE genes between the *CCDC80* and *SOD3* knockdown and control cells after Benjamini-Hochberg correction.

#### Liver HepG2 cell culture

Liver HepG2 cells (ATCC, HB-8065) were cultured in Dulbecco’s modified Eagle medium (DMEM, 31966021 Gibco: High Glucose, GlutaMAX™ Supplement, pyruvate) and supplemented with 10% fetal bovine serum (FBS, F7524, Sigma), 0.1 mM Non-Essential Amino Acids Solution (11140050, Gibco) and 1% penicillin-streptomycin. The cells were maintained at 37 °C in a humidified atmosphere at 5% CO2.

#### Treatment of liver HepG2 cells with CCDC80 and SOD3 recombinant proteins and sample collection for RNA-seq experiment

The HepG2 cells were seeded in a 12-well plate (1 × 105 cells/well) in 1 ml of culture medium. The next day, the cells were treated with *CCDC80* (Origene; Cat: TP762230) or *SOD3* (Origene; Cat: TP304156) recombinant proteins (0 and 20 ng/ml) for 24 h. Then the cells were lysed and RNA was extracted using the miRNeasy micro kit (Qiagen). Library samples were prepared using the QuantSeq 3’ mRNA-seq library prep kit FWD (Lexogen) according to the manufacturer’s instructions, amplified for 15 cycles, and then sequenced with Illumina NextSeq 500 for 75 cycles.

#### Alignment, quantification, and DE analysis of RNA-seq data from the HepG2 treatment experiment

To align and quantify the RNA-seq data from the HepG2 experiment, we utilised an identical pipeline to the one used for the siRNA knockdown experiment data (see above). We collected four replicates for each of the four experimental conditions, resulting in a total of 16 RNA-seq samples. To identify genes DE between HepG2 cells treated with *CCDC80*/*SOD3* recombinant protein and control cells, we compared the cells treated with recombinant protein (20 ng/ml) to the control cells (0 ng/ml), and we restricted the genes tested in this DE analysis to the hepatocyte marker genes which were also DE for steatosis, fibrosis, or NASH in the KOBS liver RNA-seq DE analysis (n = 61 genes tested). We assessed the significance of the DE genes between the cells treated with *CCDC80/SOD3* recombinant protein and the control cells after Benjamini-Hochberg correction (FDR < 0.05).

#### Correlation analysis of the adipose expression of the SBCs with NAFLD-related liver networks to search for associations between the SBCs and NAFLD pathways in the liver

To assess the potential connection between the adipose expression of the SBC genes and liver functions related to NAFLD, we computed the pairwise Pearson correlations between the adipose expression of every SBC and the module eigengene of each of the 10 liver co-expression networks that correlated with steatosis, fibrosis, or NASH. We assessed the significance of the correlations after correcting for multiple testing using Bonferroni correction. We then performed functional annotation analysis using WebGestalt^37^ on the liver networks that correlated with the adipose expression of the SBC genes to evaluate their biological significance.

#### Mendelian randomization analysis to search for unidirectional effects of serum triglycerides on NAFLD

To search for a unidirectional relationship between serum TGs and NAFLD, we conducted MR analysis using *cis*-eQTL data from the KOBS cohort and GWAS summary statistics from the UKB cohort.^11^ Because we hypothesised that serum TGs have a unidirectional effect on NAFLD, we considered the analysis testing for the effect of TGs on NAFLD the “forward” direction, and the analysis testing for the effect of NAFLD on TGs the “reverse” direction. Before we ran the MR analysis, we also tested for enrichment of GWAS signals for TGs and NAFLD^11^ in the *cis* regions of the adipose aware DE genes using MAGENTA,^48^ using a *cis* region definition of ±1 Mb and otherwise default parameters.

To prepare for running MR in the forward direction (impact of TGs on NAFLD), we selected a set of genetic variants to be used as IVs in the MR analysis. We began this process by identifying the *cis* regions (±1 Mb) of all adipose aware DE genes, i.e. the genes which were DE in adipose tissue but not liver in the KOBS cohort. Next, we selected DE gene *cis* regions which met the following criteria: at least one significant adipose *cis*-eQTL (FDR < 0.05) existed in the *cis* region of a gene, and at least one significant GWAS hit for TGs (p < 5 × 10^−8^) existed in the *cis* region, provided that the TG GWAS hit was not also a GWAS hit for the imputed NAFLD status (p ≥ 5 × 10^−8^). Next, we tested for colocalization in the *cis* regions passing these criteria, using the coloc.signals function of the R package coloc^49^ v5.1.0, which implements colocalization analysis that conditions on the lead variant to identify multiple signals in a single region. We used the METSIM cohort genotype data to compute the LD matrix required by coloc, using PLINK v1.9.^25^ At this point, we generated a list of IV candidates by selecting all of the colocalised *cis*-eQTL variants. To ensure the strength of our final IV list for MR analysis, we removed all IV candidates in LD with imputed NAFLD GWAS hits (r^2^ > 0.2)^11^; LD pruned IV candidates in LD with each other (r^2^ > 0.2), taking the IV with the most significant *cis*-eQTL and GWAS results as the representative signal; and removed strand-ambiguous IVs. We computed LD using PLINK v1.9,^25^ and detected strand ambiguity using the R package TwoSampleMR.^50^

With the final list of IVs, we conducted MR analysis to test for the unidirectional impact of TGs on NAFLD. To prepare the input data, we harmonised the directions of the IV effect sizes using the R package TwoSampleMR^50^ v0.5.6. Next, we ran MR using three separate methods with the default parameters: MR-PRESSO^51^ v1.0, Inverse-Variance Weighted (IVW) MR,^52^ and MR-Egger.^52^ Of these, IVW and MR-Egger are implemented in the package MendelianRandomization^52^ v0.6.0. We assessed the evidence for horizontal pleiotropy using the MR-PRESSO global test, as well as the scatterplot of IV effect sizes for TGs and NAFLD. Additionally, we ran the MR analysis using just one *VEGFB* variant, rs2845885, as a single IV. We only ran the IVW method for this single-IV analysis, due to the requirement of multiple IVs in the other three methods.

To test the reverse causal hypothesis, i.e. that NAFLD has a unidirectional effect on serum TGs, we used an identical method to the forward analysis, substituting every instance of NAFLD GWAS hits for TG GWAS hits, and vice versa. In the reverse analysis, we also used the KOBS liver aware DE genes (DE in liver but not adipose) and liver *cis*-eQTLs to select the IV candidate regions.

#### Regression analysis to determine if additional variance in NAFLD is explained by VEGFB expression when compared to serum TGs alone

To assess how much SBC adipose expression may improve the ability to explain variance in NAFLD on top of serum TGs alone, we conducted a series of regression analyses in the KOBS cohort, using *VEGFB* as the example SBC given its significant MR result (see Results). We first built logistic regression models that utilized serum TGs alone as an explanatory variable for the NAFLD status (i.e. steatosis, fibrosis, and NASH), and then built analogous models that utilized serum TGs along with the *VEGFB* adipose expression as explanatory variables for the NAFLD status (6 logistic regression models in total). Before fitting the models, we log_10_-transformed the serum TG measurements, and conducted an inverse normal transformation on the *VEGFB* expression, measured in CPM. In all regression analyses, we corrected for the same covariates that were used in the WGCNA, DE, and best subset analyses. We compared logistic regression models to each other using the Nagelkerke pseudo-r^2^ value and the area under the receiver operating characteristic curve (AUC).

Additionally, to back up the logistic regression results, we fit linear and elastic net regression models using the same explanatory and outcome variables as in the logistic regression models, as well as the same data preparation steps (6 linear models and 6 elastic net models in total). We fit the elastic net model parameters using a 10-fold cross-validation across the entire dataset. We evaluated both the linear and elastic net regression models using the r^2^ statistic, as well as the adjusted r^2^ statistic in the linear model and the coefficients of serum TGs and *VEGFB* adipose expression in the elastic net model.

### Ethics

All participants provided written informed consent to participate in this research. The KOBS (#54/2005, 104/2008, and 27/2010) and METSIM (#171/2004) studies were approved by the Ethics Committee of the Northern Savo Hospital District. The Finnish Twin study (#270/13/03/01/2008) and CRYO study (#255/13/03/01/2009) were approved by the Ethics Committee of the Hospital District of Helsinki and Uusimaa. The liver snRNA-seq study was approved by the UCLA IRB (#20–001319). All research was performed in alignment with the principles of the Helsinki Declaration.

### Statistics

The used statistical tests, justification for their use, and multiple testing correction procedures have been described in the Methods. All RNA-seq analyses were corrected for multiple testing, and the adjusted p-values < 0.05 are reported.

### Role of funders

The funders did not have any role in the study design, data collection, data analyses, interpretation, or writing of this article.

## Results

### Study design

**Fig. 1:**
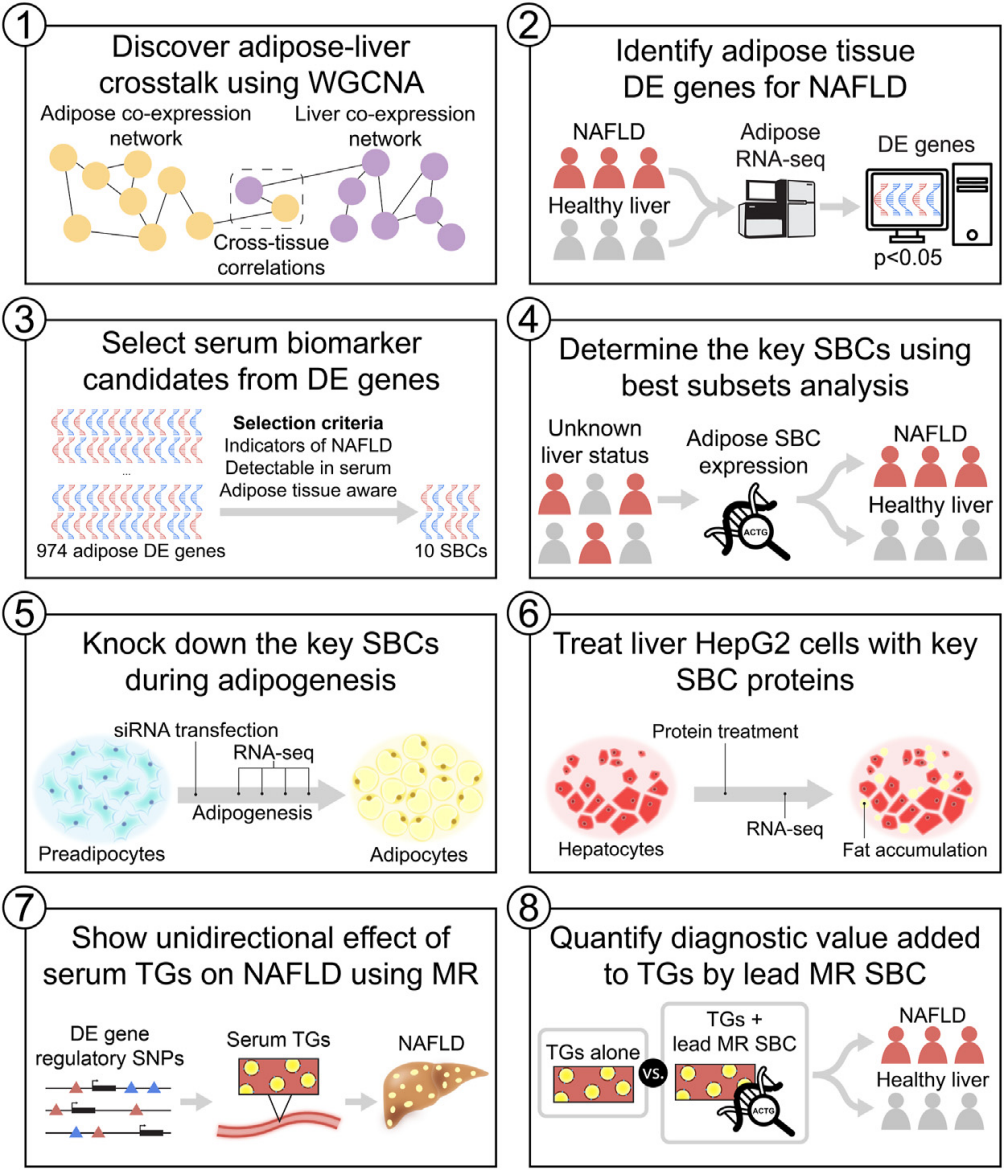
**Study design to discover 649 adipose aware differentially expressed (DE) genes, and 10 serum biomarker candidates (SBCs), for obesity-related non-alcoholic fatty liver disease (NAFLD).** To discover SBCs for obesity-related NAFLD, we leveraged a unique dual-tissue transcriptomic cohort with histology-based diagnosis of steatosis, fibrosis, and non-alcoholic steatohepatitis (NASH). 1) First, we found evidence for our hypothesis of adipose-origin NAFLD by discovering molecular crosstalk between adipose tissue and liver using WGCNA. 2) Next, we scanned genome-wide for genes DE in adipose tissue for the three NAFLD traits diagnosed by liver histology. 3) We filtered these adipose NAFLD DE genes for secreted proteins, i.e. SBCs, using a set of selection criteria, and 4) determined the key SBCs using best subset analysis. 5) We then followed up the key SBCs functionally by knocking them down in human preadipocytes during adipogenesis, and 6) treating liver HepG2 cells with their recombinant proteins. 7) Next, we demonstrated a unidirectional effect of serum triglycerides (TGs) on NAFLD using Mendelian Randomization (MR) analysis, with a set of instrumental variables (IVs) derived from the adipose aware DE genes. 8) Finally, we followed up the MR analysis by quantifying the added variance explained by the lead MR SBC in the NAFLD models in addition to serum triglycerides alone using a series of regression analyses.

We developed an integrative cross-tissue transcriptomics approach to search for genes whose changes in adipose expression reflect NAFLD in the liver (Fig. 1), leveraging a cohort of individuals with morbid obesity with RNA-seq data available from both adipose (n = 262) and liver tissue (n = 267), as well as three NAFLD traits, steatosis, fibrosis, and NASH, diagnosed by liver histology (see Methods). First, to establish that there is transcriptional crosstalk between subcutaneous adipose tissue and liver, we searched for correlations between adipose and liver co-expression networks and their associations with NAFLD. After finding evidence for crosstalk between adipose and liver expression related to NAFLD at the network level, we scanned for adipose aware DE genes, defined as genes DE in adipose tissue but not in the liver between the individuals with histology-based healthy liver and those with NAFLD. We then identified SBCs in this list of adipose aware NAFLD DE genes by selecting genes that encode for secreted proteins, are expressed highly enough in adipose tissue to be detected in serum, and are expressed substantially higher in the adipose tissue than in the liver. After selecting key SBCs from this list using best subset analysis, we investigated the functions of these SBCs in adipose tissue by knocking them down in a culture of differentiating human preadipocytes. We further investigated these key SBCs by treating human liver HepG2 cells with their recombinant proteins. We also discovered cross-tissue correlations between individual SBC genes and key liver co-expression networks correlated with NAFLD. Finally, we demonstrated a possible unidirectional effect of serum TGs on NAFLD using Mendelian Randomization analysis with a set of adipose aware NAFLD DE gene *cis*-regulatory IV variants, and found that the strongest IV regulates an SBC gene whose expression significantly adds to serum TGs in explaining variance in NAFLD.

### Co-expression networks of distinct functional pathways correlate across an individual’s subcutaneous adipose tissue and liver

To search for signatures of molecular crosstalk between adipose and liver tissue related to NAFLD, we used our dual-tissue RNA-seq cohort to construct gene co-expression networks separately in adipose and liver tissue using the R^53^ package WGCNA,^34^ and related these networks to each other (see Methods). To investigate the functional significance of the modules (i.e. networks), we correlated all adipose and liver module eigengenes with common metabolic traits and NAFLD histological liver measurements (Supplementary Fig. S1). To identify networks involved in tissue crosstalk, we correlated every adipose module eigengene with every liver module eigengene (Supplementary Fig. S1).

In agreement with our hypothesis, we found evidence that the normal physiological functions of adipose tissue, consisting of storing and burning fat, are positively associated with the normal physiological functions of liver tissue, consisting of synthesizing biomolecules into fatty acids (Supplementary Fig. S1). This was represented by positive correlation between the adipose lightyellow network (Supplementary Table S1) and the liver saddlebrown network (Supplementary Table S3) (*R* = 0.331, p = 2.296 × 10^−7^ by Pearson correlation). Adipose lightyellow is negatively correlated with serum TGs (*R* = −0.252, p = 9.979 × 10^−5^ by Pearson correlation), and is enriched for regulation of lipolysis in adipocytes (FDR = 0.0156 by WebGestalt enrichment test), insulin signalling pathways (FDR = 0.0156 by WebGestalt enrichment test), and adipocyte cell-type marker genes (p = 1.453 × 10^−7^ by hypergeometric test). Liver saddlebrown is negatively correlated with steatosis (*R* = −0.378, p = 2.572 × 10^−9^ by Pearson correlation), fibrosis (*R* = −0.306, p = 1.982 × 10^−6^ by Pearson correlation), NASH (*R* = −0.384, p = 1.302 × 10^−9^ by Pearson correlation), T2D (*R* = −0.291, p = 6.284 × 10^−6^ by Pearson correlation), and BMI (*R* = −0.277, p = 1.825 × 10^−5^ by Pearson correlation), and is enriched for the biosynthesis of amino acids pathway (FDR = 1.725 × 10^−9^ by WebGestalt enrichment test) and hepatocyte cell-type marker genes (p = 1.842 × 10^−3^, 2.202 × 10^−3^, 1.292 × 10^−4^, 7.143 × 10^−4^, and 1.513 × 10^−3^ for Hep-7, Hep-9, Hep-10, Hep-11, and Hep-13, respectively by hypergeometric test) (Supplementary Figs. S1 and S2).

Additionally, our results suggest that inflamed and dysfunctional adipose tissue is associated with a decrease in normal physiological liver function, and an increase of NAFLD and other adverse metabolic trait functions. This was represented by negative correlation between the adipose cyan network (Supplementary Table S2) and liver saddlebrown (*R* = −0.351, p = 3.561 × 10^−8^ by Pearson correlation). Adipose cyan is positively correlated with NASH (*R* = 0.268, p = 3.303 × 10^−5^ by Pearson correlation) (Supplementary Fig. S1), and is enriched for autoimmune and inflammatory pathways, including inflammatory bowel disease (FDR = 1.824 × 10^−11^ by WebGestalt enrichment test) and autoimmune thyroid disease (FDR = 5.558 × 10^−13^ by WebGestalt enrichment test) (Supplementary Fig. S2b).

Taken together, our gene co-expression network results indicate that there are correlated networks between an individual’s adipose tissue and liver, and that these normal physiological correlations are significantly inverted in NAFLD. To investigate the details of this tissue crosstalk, we aimed to move past the co-expression network level and discover individual genes as indicators for adipose dysfunction-related NAFLD.

### A total of 649 genes are DE in subcutaneous adipose tissue, but not in the liver, between individuals with and without NAFLD

**Fig. 2:**
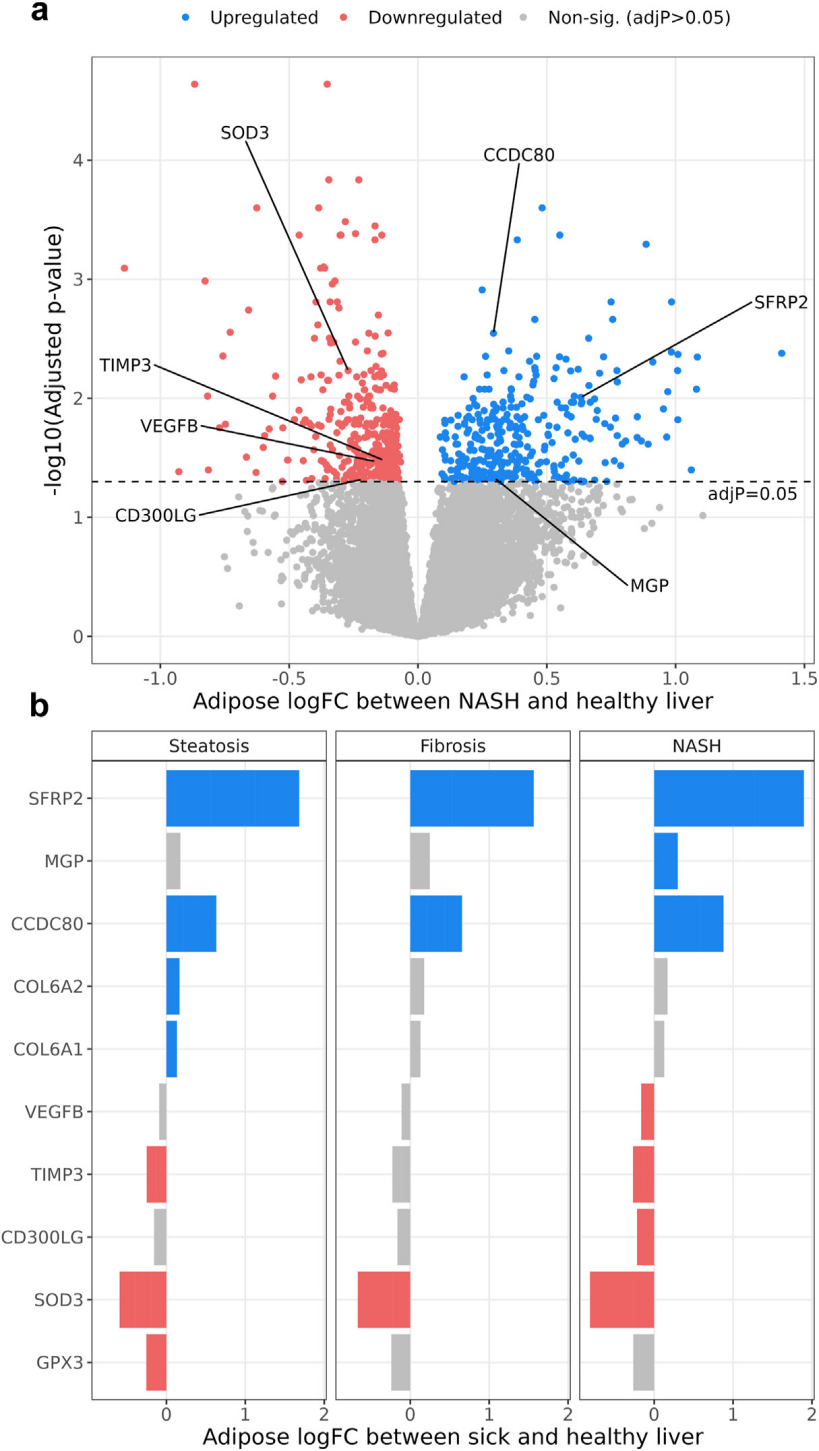
**A total of 953 genes are differentially expressed (DE) in subcutaneous adipose tissue between the obese individuals with the three main non-alcoholic fatty liver disease (NAFLD) traits, steatosis, fibrosis and/or non-alcoholic steatohepatitis (NASH), and the obese individuals with healthy livers.** We performed DE analysis on bulk RNA-seq data from subcutaneous adipose biopsies in the KOBS cohort, comparing individuals with the NAFLD traits diagnosed by liver histology to those with healthy livers. Gene counts represent numbers of genes DE for NAFLD in the subcutaneous adipose tissue before filtering for serum biomarker candidates (SBCs). Of the 953 adipose DE genes, 680, 273, and 663 genes are DE for steatosis, fibrosis, and NASH, respectively. (a) Volcano plot showing the results of the NASH DE analysis in the adipose tissue. The X-axis represents log fold-change (logFC) in adipose bulk RNA-seq data from individuals with NASH and those with healthy livers. The Y-axis represents the negative log of the DE p-value, adjusted for multiple testing with the Benjamini-Hochberg procedure. Significant SBCs identified in our subsequent filtering steps (Fig. 3) are highlighted. Volcano plots of steatosis and fibrosis DE results in the subcutaneous adipose tissue are shown in Supplementary Fig. S3. (b) Bar plot showing the DE direction of the SBCs in the adipose DE analysis for steatosis, fibrosis, and NASH. X-axis represents logFC in adipose bulk RNA-seq data from individuals with each NAFLD trait and those with healthy livers. Y-axis represents the SBC name, sorted by logFC. Blue SBCs have increased adipose expression in individuals with NAFLD when compared to the individuals with healthy livers, while red SBCs have decreased adipose expression.

Given the observed correlations between adipose and liver co-expression networks, we hypothesised that there would be adipose-liver crosstalk mediated by proteins secreted by adipose tissue that affect NAFLD. To this end, we first identified genes whose adipose expression was associated with three key histology-based NAFLD traits (steatosis, fibrosis, and NASH), using DE analysis with the R limma-voom pipeline.^40^ We compared the adipose expression of individuals with steatosis, fibrosis, and NASH to those with healthy livers, while correcting for common demographic and technical confounders (see Methods). We identified 953 genes DE for at least one NAFLD histology trait (680, 273, and 663 DE genes for steatosis, fibrosis, and NASH, respectively) (Fig. 2, Supplementary Fig. S3 and Table S8). All 953 DE genes were determined after adjusting for multiple testing with the Benjamini-Hochberg method, and thus we used adjusted p < 0.05 (i.e. FDR < 0.05) for determining the significance of the DE genes. To select genes with adipose tissue aware differential expression, we filtered out all liver DE genes for the same three NAFLD traits (Supplementary Table S9). This resulted in 649 total adipose aware DE genes (440, 188, and 471 adipose aware DE genes for steatosis, fibrosis, and NASH, respectively). As expected, these adipose aware DE genes were enriched in both the adipose lightyellow (p = 1.467 × 10^−7^ by hypergeometric test) and adipose cyan (p = 6.430 × 10^−13^ by hypergeometric test) adipose co-expression networks (Supplementary Tables S1 and S2), in line with our hypothesis that dysfunctional adipose tissue is associated with NAFLD. Next, we focused on the genes among these 649 that were most likely to be detectable in serum to discover potential adipose-origin serum biomarkers for NAFLD.

### Identification of 10 SBCs for NAFLD

**Fig. 3:**
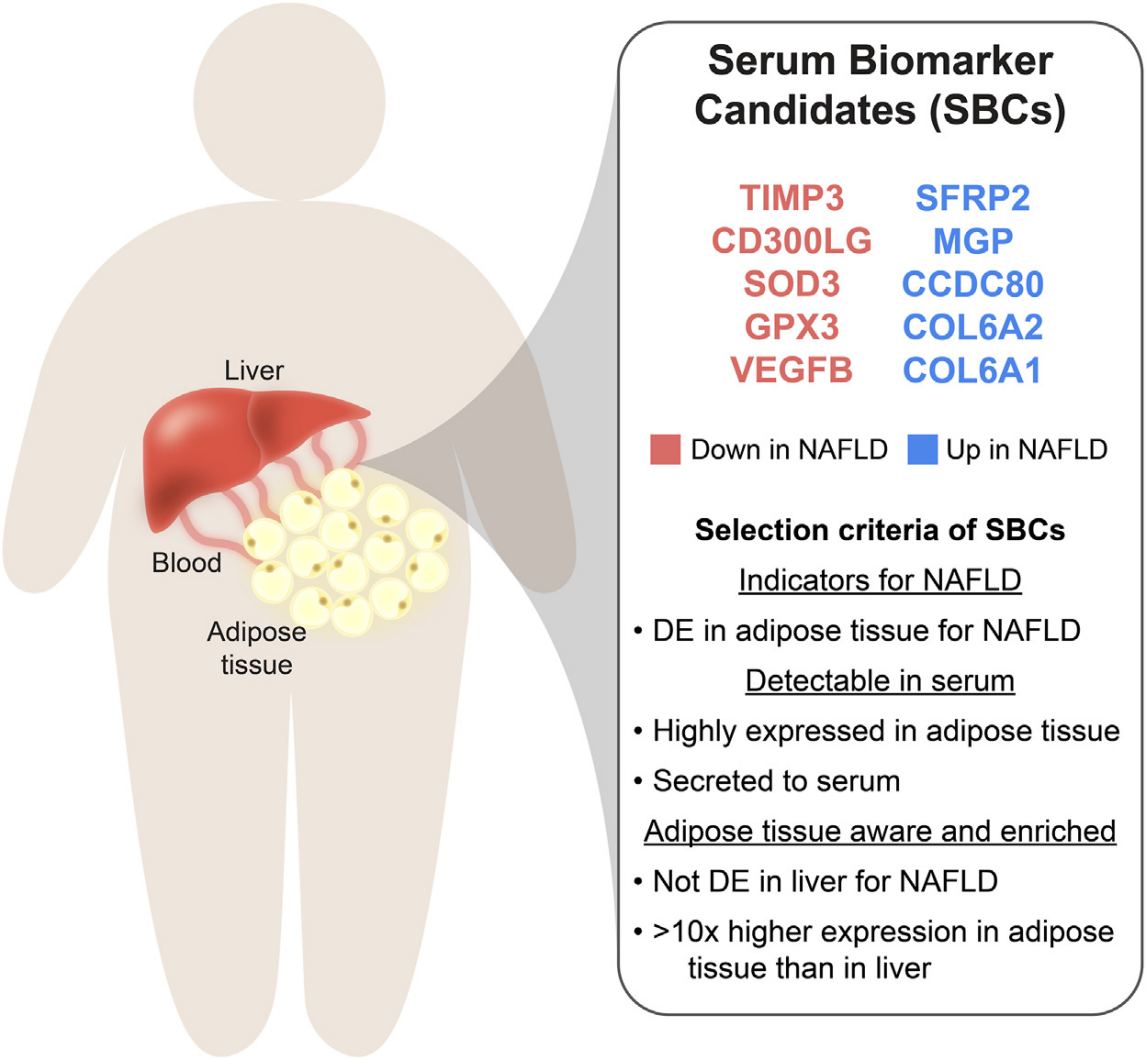
**Filtering of subcutaneous adipose non-alcoholic fatty liver disease (NAFLD) differentially expressed (DE) genes to select serum biomarker candidates (SBCs).** To identify SBCs among the list of 953 adipose NAFLD DE genes, we selected the genes that were DE for NAFLD in adipose tissue but not in the liver, coded for secreted proteins, had moderate to high expression in adipose tissue, and had >10x higher expression in the subcutaneous adipose tissue than in the liver. These filters reduced the list of 953 total adipose DE genes across steatosis, fibrosis, and non-alcoholic steatohepatitis (NASH) to 10 SBCs. Blue genes are upregulated in steatosis, fibrosis, and/or NASH in adipose tissue, while red genes are downregulated.

We reasoned that the adipose aware NAFLD DE genes that are effective SBCs must leave the cell, be expressed at sufficient levels in their source tissue to be detectable in serum, and have predominantly adipose enriched expression. To implement these constraints, we filtered the 649 adipose aware DE genes for the ones present in the Human Protein Atlas (HPA)^41^ list of secreted proteins, and with median TPM greater than 30 in subcutaneous adipose tissue, using data from the GTEx portal. Additionally, we filtered out all genes whose ratio of subcutaneous adipose median TPM to liver median TPM was less than 10. This design resulted in a final list of 10 SBCs: *CCDC80*, *CD3*00LG, *COL6A1*, *COL6A2*, *GPX3*, *MGP*, *SFRP2*, *SOD3*, *TIMP3*, and *VEGFB* (Fig. 3). Taken together, all SBCs are DE in subcutaneous adipose tissue for at least one NAFLD trait (steatosis, fibrosis, or NASH), are not DE in liver for any of the same three NAFLD traits, code for secreted proteins, have median TPM > 30 in subcutaneous adipose tissue, and have >10x higher median TPM in subcutaneous adipose tissue than in liver tissue.

### Determination of key SBCs using best subset modelling approach

**Fig. 4:**
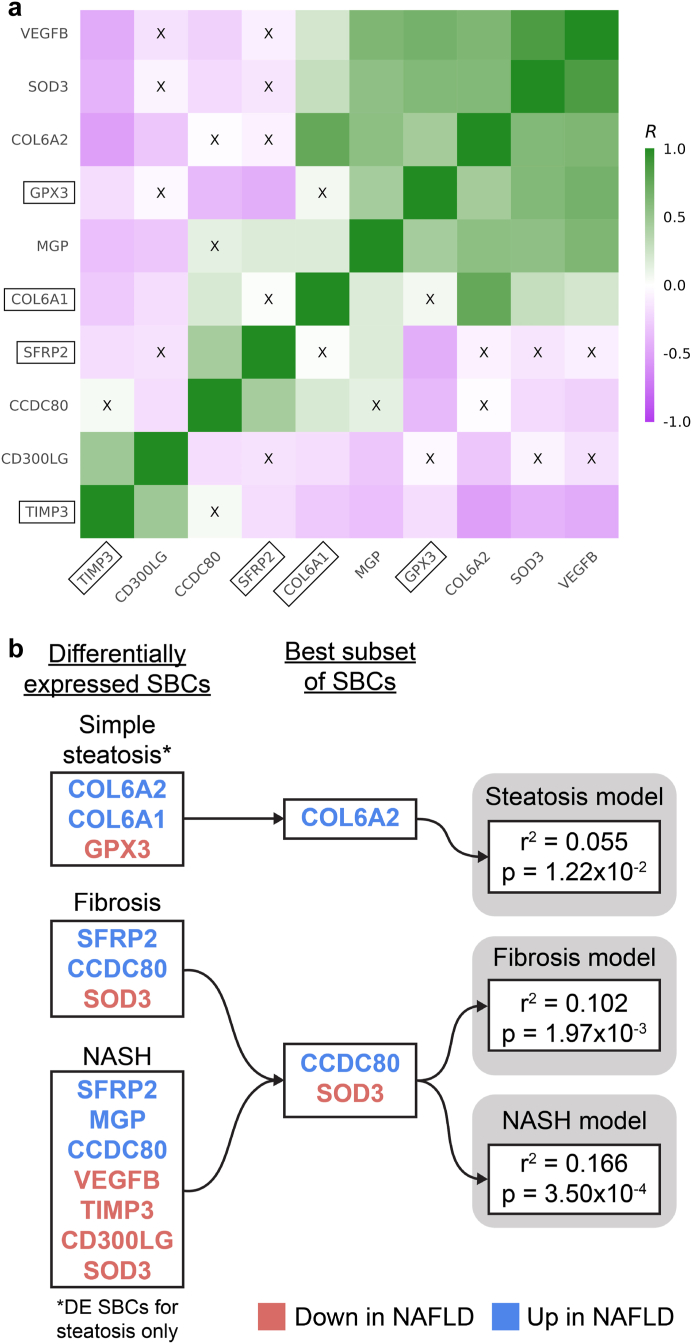
**Selection of the key serum biomarker candidates (SBCs) using the best subset analysis, motivated by our prior gene-gene correlations observed in the adipose expression of the SBCs.** We filtered the list of 10 SBCs further by testing the proportion of variance explained in steatosis, fibrosis, and non-alcoholic steatohepatitis (NASH) by the adipose expression of the SBCs. (a) Pairwise gene-gene correlation structure between the subcutaneous adipose expression of the SBC genes. Each coloured box represents the strength of the pairwise Pearson correlation (*R*) between the adipose expression of the SBC genes. Green boxes correspond to a positive correlation, and purple boxes correspond to a negative correlation. “X” indicates that the correlation is non-significant after Bonferroni correction. Genes are ordered by the first principal component (PC). Boxed gene names represent the SBCs that correlate with the key NAFLD-related liver network module eigengenes. The observed correlations between the adipose expression of the SBCs motivate the idea that a small subset of the SBCs can capture most of the expression of all 10 SBCs, which we then tested in the best subset analysis. (b) Results of the best subset analysis. For steatosis, fibrosis, and NASH, the best subset of significant SBCs was chosen by the leaps algorithm, based on the variance in the non-alcoholic fatty liver disease (NAFLD) trait explained by each combination of genes. P-values were calculated based on a permutation test (B = 100,000) (see Methods). To capture genes involved in the early onset of NAFLD, only the 3 genes that were uniquely differentially expressed (DE) for steatosis in the subcutaneous adipose tissue were considered for the steatosis model.

To find the best subset of these 10 genes for evaluating NAFLD risk, we modelled the effect of their adipose expression on NAFLD. First, we observed that there are significant adipose expression gene-gene correlations among the 10 SBCs (Fig. 4a), indicating that they are not fully independently expressed in the adipose tissue. To avoid redundancy, we then searched for the minimum set among the 10 SBCs whose adipose expression explained the maximum amount of variance in NAFLD, using a best subset approach with the R package leaps.^43^ In this approach, we fit linear models for different combinations and numbers of SBCs, and tested the variance in steatosis, fibrosis, and NASH explained by each combination of genes, while correcting for the same covariates we used in the WGCNA and DE analyses (see Methods). We discovered that the gene Collagen Type VI Alpha 2 Chain (*COL6A2*) explains the most variation in steatosis (r^2^ = 0.055, p_permutation_ = 1.22 × 10^−2^ by permutation test), and the genes *CCDC80* and *SOD3* explain the most variation in both fibrosis and NASH (r^2^ = 0.102, p_permutation_ = 1.97 × 10^−3^ for fibrosis; r^2^ = 0.166, p_permutation_ = 3.50 × 10^−4^ for NASH, by permutation test) (Fig. 4b, Supplementary Table S10). This result further strengthens the premise of *CCDC80* and *SOD3* as biomarkers, because our permutation results show that their adipose expression explains more variation in fibrosis and NASH than virtually all other pairs of genes.

### Effect of CCDC80 and SOD3 knockdown on human preadipocytes during adipogenesis

**Fig. 5:**
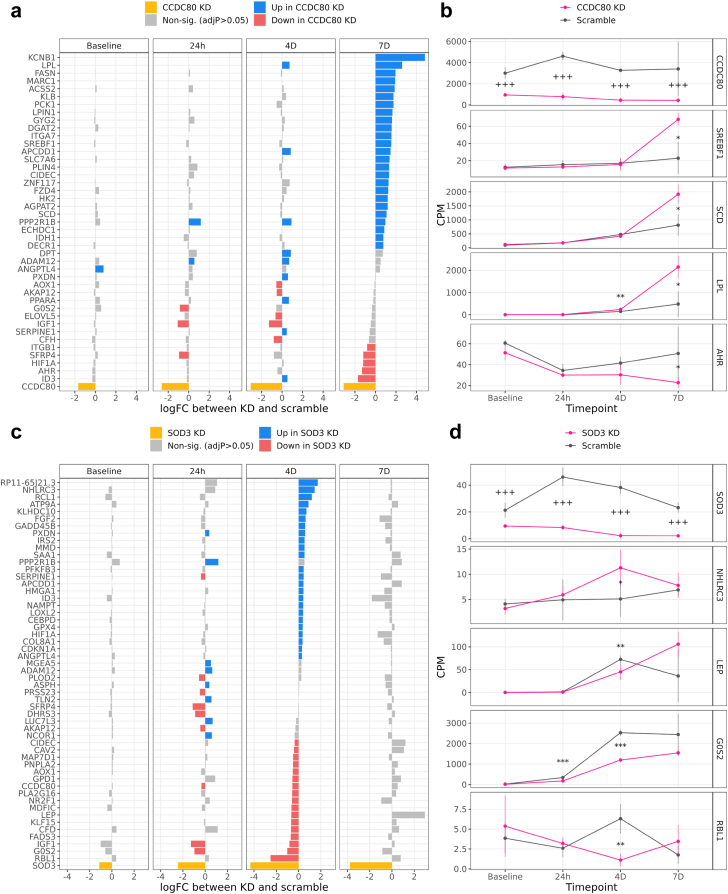
***CCDC80* knockdown in human preadipocytes differentiated to adipocytes activates known drivers of adipogenesis, and *SOD3* knockdown deactivates known drivers of healthy energy homeostasis.** We knocked down *CCDC80* and *SOD3* using siRNA transfection in independent cultures of human SGBS preadipocyte cells (see Methods), and measured expression via RNA-seq at 4 time points during adipogenesis. We then performed a differential expression (DE) analysis on the RNA-seq data between the *CCDC80* or *SOD3* gene knockdown and scramble conditions at each time point. (a) Results of the *CCDC80* knockdown DE analysis. The X-axis represents the log fold-change (logFC) of all 43 genes which were DE in at least one time point during the differentiation; the Y-axis the gene names; and facets the time points of adipogenesis. Blue genes were expressed significantly more in the *CCDC80* knockdown than in the scramble conditions, and red genes were expressed less. Yellow represents *CCDC80*, the knocked down gene. (b) Mean expression of *CCDC80* and selected well known examples of adipogenesis genes in the scramble and knockdown samples during differentiation. The X-axis represents the time point of the adipocyte differentiation; the Y-axis counts per million (CPM); facets the gene name; error bars the mean ± one standard deviation; and colours the experimental condition (knockdown or scrambled control). Each condition-timepoint combination within each facet represents n ≥ 3 samples, and the total experiment included n = 28 samples. Annotations indicate the significance of DE between the knockdown and scramble samples for a given timepoint and gene: “∗∗∗” = adjP < 0.001; “∗∗” = adjP < 0.01; “∗” = adjP < 0.05. In the *CCDC80* panel only: “+++” = p < 0.001, “++” = p < 0.01, “+” = p < 0.05. The *CCDC80* p-values are not adjusted for multiple testing because we directly manipulated *CCDC80* expression in the knockdown experiment. (c) Results of the *SOD3* DE analysis, with 54 DE genes. Plot elements are analogous to those in (a). (d) Mean expression of *SOD3* and selected well known examples of adipogenesis and satiety signalling genes in the knockdown and scramble samples during differentiation. Plot elements are analogous to those in (b).

Because *CCDC80* and *SOD3* were observed as the strongest SBCs for both fibrosis and NASH, we next investigated their effects on adipogenesis *in vitro* using an siRNA knockdown experiment. In this experiment, we cultured human SGBS preadipocytes over the course of differentiation to adipocytes, and collected bulk RNA-seq data at four time points. We first confirmed that the knockdown was effective, as evidenced by the downregulation of both *CCDC80* and *SOD3* (p < 0.05) in their respective knockdown conditions (Fig. 5, Supplementary Tables S11 and S12). We then performed Oil Red O (ORO) staining on the SGBS preadipocytes at each time point of adipogenesis to compare the amount of lipid accumulation between the scrambled control and *CCDC80* and *SOD3* knockdown samples (Supplementary Fig. S4). We discovered that there are significant changes in the ORO intensity in both the *CCDC80* and *SOD3* knockdowns during adipogenesis when compared to the scrambled control. By day 4, the ORO intensity was significantly decreased in both knockdowns when compared to the scrambled control, and by day 7, there was either no significant difference (*CCDC80*), or the knockdown samples had significantly higher intensity (*SOD3*) than the scrambled control (Supplementary Fig. S4). The ORO intensity values of the scrambled control did not significantly differ from the ones in the non-scrambled control in these experiments (all P > 0.05 by Student’s t-test). As the ORO intensity can quantify the amount of fat present in the sample, this evidence suggests that knocking down *CCDC80* and *SOD3* delays the onset of adipogenesis.

Next, we searched for DE genes between control and separate knockdown of *CCDC80* and *SOD3* at each adipogenesis time point from baseline to seven days (see Methods). Because we were interested in the impact of *CCDC80* and *SOD3* knockdown on adipogenesis specifically, we restricted the genes tested for DE to a list of preadipocyte, adipocyte, and adipogenesis marker genes (n = 492 genes tested, see Methods).

We found evidence suggesting that *CCDC80* may contribute to NAFLD progression by inhibiting the ability of adipose tissue to produce new adipocytes to store fat. This was supported first by the observation that *CCDC80* adipose expression is increased in the obese subjects with NAFLD when compared to the obese individuals with healthy liver (Fig. 2, Supplementary Table S8), and second, by the observation that knockdown of *CCDC80* during adipogenesis significantly increased the expression of fatty acid master transcription factor *SREBF1* at 7 days (log fold-change in knockdown compared to control (logFC) = 1.547, p = 8.608 × 10^−4^ *vs* control by R package limma) as well as TG hydrolysis enzyme Lipoprotein Lipase (*LPL*) at 7 days (logFC = 2.597, p = 7.215 × 10^−4^ *vs* control by R package limma) (Fig. 5a and b, Supplementary Table S11). Of the 141 adipogenesis pathway genes we downloaded from WikiPathways, 13 were DE during at least one timepoint in the *CCDC80* knockdown (Fig. 5a and b, Supplementary Table S11).

We also found evidence suggesting that *SOD3* may protect against NAFLD by promoting a healthy satiety feedback loop. This was supported first by the observation that *SOD3* adipose expression is decreased in individuals with NAFLD compared to those with healthy livers (Fig. 2, Supplementary Table S8), and second, by the observation that the knockdown of *SOD3* during adipogenesis significantly decreased the expression of the satiety signalling protein *LEP* at 4 days (logFC = −0.651, p = 1.966 × 10^−4^ *vs* control by R package limma) (Fig. 5c and d, Supplementary Table S12). Seventeen of the adipogenesis pathway genes were DE during at least one timepoint in the *SOD3* knockdown (Fig. 5c and d, Supplementary Table S12).

### Effect of CCDC80 and SOD3 recombinant proteins on human liver HepG2 cells

To further explore the potential functional role of the key SBCs *CCDC80* and *SOD3* in NAFLD, we treated liver HepG2 cells separately with *CCDC80* and *SOD3* recombinant protein for 24 h. To assess the changes induced by the treatments with the *CCDC80* and *SOD3* recombinant proteins, we performed RNA-seq on the treated and non-treated HepG2 cells, and then conducted a DE analysis separately comparing the *CCDC80* and *SOD3* treated HepG2 cells to the non-treated control HepG2 cells (see Methods). Because we were interested in the impact of the *CCDC80* and *SOD3* recombinant proteins on processes related specifically to the development of NAFLD in liver cells, we restricted the tested genes to the hepatocyte marker genes which were also DE for steatosis, fibrosis, or NASH in the KOBS liver RNA-seq DE analysis (n = 61 genes tested).

**Fig. 6:**
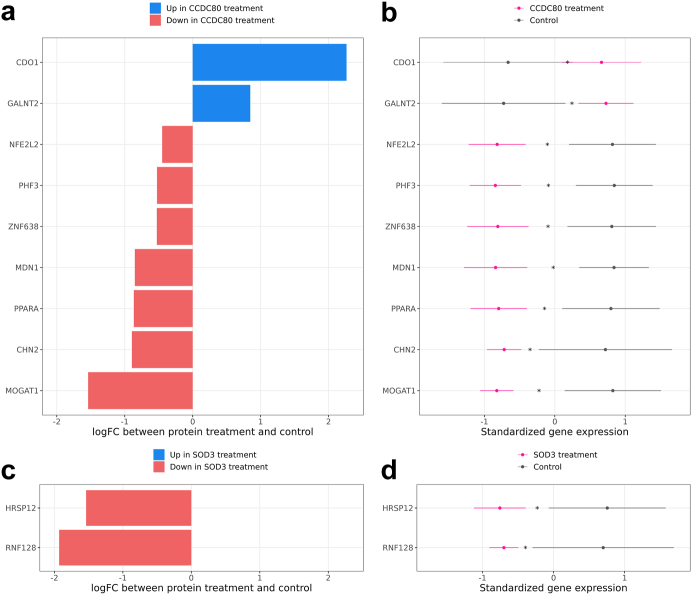
**Treatment of human liver HepG2 cells with the *CCDC80* and *SOD3* recombinant proteins changes the expression of several known NAFLD-related genes.** We treated separate cultures of human liver HepG2 cells with the *CCDC80* and *SOD3* recombinant proteins for 24 h (see Methods), and measured expression via RNA-seq. We then performed a differential expression (DE) analysis between the recombinant protein treated cells and non-treated control cells. (a) Results of the *CCDC80* treatment DE analysis. The X-axis represents the log fold-change (logFC) of the 9 significant DE genes, and the Y-axis represents the gene names. Blue genes were expressed significantly more in the *CCDC80* treated cells than in the control cells, and red genes were expressed less. (b) Mean expression of all 9 significant DE genes in the *CCDC80* treated cells compared to the control cells. The X-axis represents counts per million (CPM), standardized by the mean and standard deviation of each gene; the Y-axis gene name; error bars the mean ± one standard deviation; and colours the experimental condition (*CCDC80* treated HepG2 cells or non-treated control cells). Each row represents 4 samples treated with *CCDC80* and 4 control samples, for a total of 8 samples per row. The “∗” annotation indicates that the gene was significantly DE between the *CCDC80* treatment and control samples (adjP < 0.05 after Benjamini–Hochberg correction (FDR < 0.05)). (c) Results of the *SOD3* treatment DE analysis, with 2 significant DE genes. Plot elements are analogous to those in (a). (d) Mean expression of the 2 DE genes in the *SOD3* treated cells compared to the control cells. Plot elements are analogous to those in (b).

In the DE analysis of the 61 genes, we identified a total of 11 DE genes with significant, multiple testing corrected p-values (FDR < 0.05), 9 for *CCDC80* and 2 for *SOD3* (Fig. 6, Supplementary Table S13). Among these 11 genes, we highlight three genes, Peroxisome Proliferator Activated Receptor Alpha (*PPARA*), NFE2 Like BZIP Transcription Factor 2 (*NFE2L2*) (also called *NRF2*), and Ring Finger Protein 128 (*RNF128*) (also called *GRAIL*), with ample previous evidence in liver fatty acid metabolism and fat accumulation in hepatocytes (see Discussion). Our experiment treating liver HepG2 cells with the *CCDC80* recombinant protein shows that the *PPARA* and *NFE2L2* genes had a significantly lower expression in the *CCDC80* treated HepG2 cells when compared to the control HepG2 cells (logFC = −0.866, −0.449, p = 2.141 × 10^−3^, 7.057 × 10^−4^ *vs* control cells, by R package limma for *PPARA* and *NFE2L2*, respectively). We also show that the *RNF128* gene had a significantly lower expression in the *SOD3* treated HepG2 cells when compared to the control HepG2 cells (logFC = −1.930, p = 1.652 × 10^−3^ *vs* control cells, by R package limma) (Fig. 6, Supplementary Table S13). Taken together with the previously published NAFLD-related evidence for these three genes (see Discussion) our results suggest that the *CCDC80* protein may induce hepatocytes to accumulate fat, while the *SOD3* protein may promote healthy lipid metabolism in hepatocytes, in line with the observed increased (*CCDC80*) and decreased (*SOD3*) adipose expression in individuals with NAFLD (Fig. 2).

### Adipose expression of four SBCs correlates with key liver co-expression networks associated with NAFLD

To investigate how individual SBC genes may be connected to liver functions related to NAFLD, we correlated the adipose expression of every SBC with the module eigengene of each of the 10 liver networks that correlated with steatosis, fibrosis, or NASH (i.e. the liver saddlebrown, cyan, tan, magenta, lightcyan1, lightgreen, darkgrey, violet, darkmagenta, and royalblue network modules) (Supplementary Fig. S1 and Table S14). We found that the adipose expression of four SBCs, *GPX3*, *COL6A1*, *SFRP2*, and *TIMP3*, correlates significantly with the key NAFLD-associated liver networks, which represent important functional liver pathways, including the biosynthesis of amino acids, sugar and fatty acid metabolism, and metabolic processes (Supplementary Figs. 2c–h and Table S14).

Notably, we also found that the adipose expression of these genes, *GPX3*, *COL6A1*, *SFRP2*, and *TIMP3*, seems to be effective at capturing the adipose expression of all 10 SBCs, as demonstrated by their membership in each of the significant (passing multiple testing correction) gene-gene correlation blocks in Fig. 4a. In more detail, Fig. 4a shows that *GPX3* belongs to the significant correlation block with *VEGFB*, *SOD3*, *COL6A2*, *GPX3*, and *MGP*; *SFRP2* belongs to the significant correlation block with *CCDC80*; *COL6A1* forms a singleton correlation block with itself as it is not significantly correlated with other SBCs; and *TIMP3* belongs to the significant correlation block with *CD3*00LG and *TIMP3*. Thus, we can conclude that the adipose expression of these 4 SBCs, which represent all positively correlated gene-gene correlation blocks among the 10 SBC genes in Fig. 4a, are significantly correlated with the NAFLD-associated liver networks, and in turn with numerous co-expressed liver genes.

### TG GWAS variants regulating the adipose NAFLD DE genes in cis help identify a unidirectional pathway from serum TGs to NAFLD

**Fig. 7:**
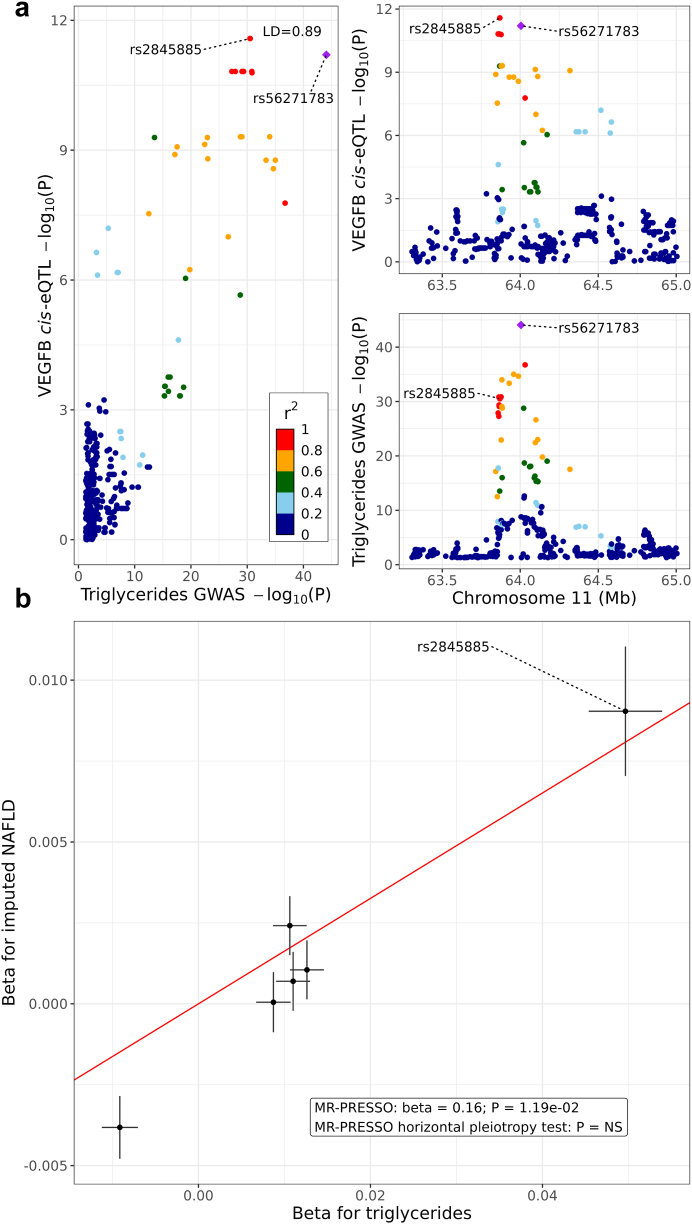
**Mendelian Randomization (MR) analysis suggests a unidirectional effect of serum triglycerides (TG) on imputed non-alcoholic fatty liver disease (NAFLD) status, mediated by *cis* regulators of adipose aware DE genes.** We derived 6 instrumental variable (IV) variants for MR analysis from the *cis* regions of adipose aware differentially expressed (DE) genes by selecting adipose *cis*-expression quantitative trait loci (*cis*-eQTL) SNPs colocalised with TG GWAS SNPs that were not also NAFLD genome-wide association study (GWAS) SNPs. We then conducted MR analysis with MR-PRESSO using variant GWAS effect sizes from the UK Biobank, and discovered a significant result. (a) Colocalization of *VEGFB* adipose *cis*-eQTL rs2845885 with TG GWAS variant rs56271783. Strong colocalization of variants regulating both TGs and adipose expression of an SBC gene suggests that key DE genes and serum TG levels may share a directional pathway. Each point represents one genetic variant, and colour indicates pairwise linkage disequilibrium (LD) with rs56271783, as described in the legend in the left panel. Upper right panel: X-axis represents position on chromosome 11 in megabases (Mb). Y-axis represents the significance of variant association with *VEGFB* adipose expression, i.e. the negative log p-value from adipose *cis*-eQTL analysis. Bottom right panel: X-axis represents the position on chromosome 11 in megabases (Mb). Y-axis represents the significance of variant association with serum TG levels, i.e. the negative log P-value from GWAS analysis. Left panel: X-axis represents the negative log p-value from TG GWAS analysis. Y-axis represents the negative log P-value from *VEGFB* adipose *cis*-eQTL analysis. Annotation reports the LD value between rs2845885 and rs56271783. (b) Results of MR analysis with MR-PRESSO. The absence of outliers in the plot indicates that there is no significant horizontal pleiotropy in the set of IVs, as evidenced by non-significance in the MR-PRESSO global test. Each point represents an IV, and error bars represent the effect size ± SE. X-axis represents the variant effect size for serum TGs, while Y-axis represents the variant effect size for the imputed NAFLD status. Regression line is generated from the MR-PRESSO output slope with an intercept of 0.

To understand the role of the adipose aware NAFLD DE genes in NAFLD pathogenesis, we conducted MR analysis using a set of IVs derived from adipose aware NAFLD DE gene *cis*-expression quantitative trait loci (*cis*-eQTLs) and TG genome-wide association study (GWAS) results. We reasoned that it is important to search for specific biological pathways that cause adipose dysfunction to impact serum TGs, and in turn drive NAFLD onset in the liver, because TG measurements alone are not sufficient to diagnose NAFLD. Elevated TG levels can be impacted by a wide variety of different genetic and environmental factors, including but not limited to adipose dysfunction and NAFLD. To select our set of IV variants, we began with the adipose aware NAFLD DE genes (n = 649 genes), motivated by the fact that we observed a significant enrichment of TG GWAS variants in their *cis* regions (±1 Mb) (FDR_95_ = 8.00 × 10^−4^, FDR_75_ = 4.4 × 10^−3^ by MAGENTA enrichment test), but not for NAFLD GWAS variants (FDR > 0.05) using MAGENTA. Of the 649 adipose aware NAFLD DE genes, we selected those with a significant adipose *cis*-eQTL in the KOBS bulk RNA-seq data and a TG GWAS hit that was not a NAFLD GWAS hit in their *cis* regions (n = 191 valid *cis* regions). We tested for colocalization between *cis*-eQTLs and TG GWAS variants in each candidate region (n = 10 significantly colocalised regions, with the strongest colocalised signal shown in Fig. 7a), selected the colocalised *cis*-eQTLs as IV candidates, LD pruned the IV candidates, and removed strand ambiguous IVs (n = 6 final IVs, see Methods). We ran 3 methods of MR analysis with these 6 IVs, using the UKB GWAS summary statistics for TGs and NAFLD (see Methods) as the input data.

Using MR analysis, we discovered evidence of a unidirectional positive effect of serum TGs on NAFLD, as first demonstrated by a significant result in the MR-PRESSO method (Beta = 0.163, p = 1.188 × 10^−2^ by MR-PRESSO global test). Based on the MR-PRESSO global test, there was no horizontal pleiotropy among the 6 IVs (p > 0.05), and there were no outliers in effect-size space (Fig. 7b). We found a similar significant result with both the IVW MR method (Beta = 0.162, p = 1.284 × 10^−4^ by Wald test), and the MR-Egger method (Beta = 0.180, p = 0.036 by Wald test).

Notably, our MR results also suggest that the SBC Vascular Endothelial Growth Factor B (*VEGFB*) (Fig. 7a) may belong to a biological pathway upstream of NAFLD. This is supported by the fact that one of the adipose IVs, rs2845885, is an adipose but not liver *cis*-eQTL for *VEGFB* (Beta_*cis*-eQTL_ = −0.722, FDR_*cis*-eQTL_ = 8.833 × 10^−10^ by Matrix eQTL association test). By our IV definition, rs2845885 is also a significant GWAS variant for TG (Beta_GWAS_ = 0.050, p_GWAS_ = 2.9 × 10^−31^ by BOLT-LMM GWAS test), and not a significant GWAS variant for NAFLD. The single nucleotide polymorphism (SNP) rs2845885 is significantly colocalised with the TG GWAS variant rs56271783 (posterior probability of colocalisation = 98.8%) (Fig. 7a). When run by itself as the sole IV in the IVW MR method, which can be run for single variants, rs2845885 still produces a significant result for the path from serum TGs to NAFLD (Beta_MR_ = 0.182, p_MR_ = 6.153 × 10^−6^ by Wald test). This indicates that the SBC, *VEGFB*, likely plays a significant role in the observed effect of serum TGs on NAFLD that is induced by obesity-related adipose dysfunction.

To ensure our MR evidence indicated an adipose-origin unidirectional effect of TGs on NAFLD, we verified that the reverse causal hypothesis, i.e. that NAFLD drives changes in serum TG levels, lacked substantive evidence. To select IVs for the reverse direction MR analysis, we first selected liver aware DE genes, which were DE in the liver but not in the adipose tissue (n = 304 genes), selected those with a significant liver *cis*-eQTL and a NAFLD GWAS hit that was not also a TG GWAS hit in their *cis* regions (n = 1 valid *cis* region), and tested those for colocalization in the single remaining *cis* region of the NEDD4 Like E3 Ubiquitin Protein Ligase (*NEDD4L*) gene (see Methods). There was no significant colocalization in this *NEDD4L* gene region, meaning that there were zero valid IVs for the reverse MR analysis. Overall, this suggests that changes in adipose tissue function may influence NAFLD via serum TGs, rather than the other way around.

### Adipose expression of the lead MR SBC, VEGFB, explains additional variance in NAFLD compared to serum TGs alone

To further explore the MR results, implying that *VEGFB* may contribute to the measured effect of TGs on NAFLD, we quantified the potential diagnostic value of *VEGFB* adipose expression in comparison to TG measurements alone using a series of regression analyses. We first fit two types of logistic regression models to the steatosis, fibrosis, and NASH status in the KOBS cohort, while correcting for the same covariates as we did in the DE and best subset analyses. The first model utilised only serum TGs as an explanatory variable, and the second added adipose *VEGFB* expression to the model, utilising both TGs and adipose *VEGFB* expression as the explanatory variables for the NAFLD status (steatosis, fibrosis, and NASH). We found that adding *VEGFB* expression to the model substantially increased the goodness of fit over the serum TGs alone. In more detail, the NASH model showed the strongest improvement, in which the pseudo-r^2^ increased from 23.4% to 27.8%, and the area under the receiver operating characteristic curve (AUC) increased from 73.7% to 76.6%, after the addition of the expression of just one SBC, *VEGFB* (Supplementary Table S15). We observed similar increases in fibrosis, and slightly smaller increases in steatosis.

To further confirm these results, we then built linear and elastic net models with identical combinations of explanatory variables, and found that, as expected given the logistic regression results described above, adding *VEGFB* increased the goodness of fit across all model types and NAFLD phenotypes over the serum TGs alone. This result was preserved in the adjusted r^2^ statistic from the linear model, and *VEGFB* also had a nonzero coefficient in the elastic net models for fibrosis and NASH, indicating that *VEGFB* expression contributes significantly to the model (Supplementary Tables S16 and S17).

## Discussion

We hypothesised that in some obese individuals, obesity induces pathological inflammatory changes in the subcutaneous adipose tissue, leading to ectopic deposition of fat into the liver and the development of NAFLD, and that these mechanisms could be observed in the changes in adipose expression of key genes related to adipose tissue function. Eventually, these pathways could also be traced without liver biopsy or abdominal imaging using adipose origin SBCs. To test our hypothesis, we first demonstrated that there may be crosstalk between the adipose tissue and liver by identifying correlations between adipose and liver gene co-expression networks associated with NAFLD and related metabolic traits. Next, we identified 649 adipose tissue aware DE genes for liver histology-based NAFLD phenotypes in individuals with morbid obesity with and without NAFLD. Filtering these adipose aware DE genes resulted in the identification of 10 SBCs, which are DE in adipose, are not DE in the liver, show adipose enriched expression, and encode proteins secreted to serum. Based on best subset analysis, we further follow-up the SBCs, *CCDC80* and *SOD3*, by knockdown in human preadipocytes and subsequent differentiation experiments, which suggest that their knockdown impacts important adipogenesis genes. Finally, the TG GWAS variants regulating the adipose NAFLD DE genes in *cis* helped us identify a possible unidirectional pathway from serum TGs to NAFLD.

Previous work has utilised transcriptomics data and direct serum protein measurements paired with NAFLD diagnosis to search for noninvasive biomarkers for NAFLD.^2^^,^^9^ However, to the best of our knowledge, our study may be the first to leverage a dual-tissue cohort with both adipose tissue and liver RNA-seq available, paired to a gold-standard NAFLD diagnosis using liver histology. The originality of our study lies not primarily in our hypothesis, rather in our approach to identify cross-tissue disease effects using dual-tissue omics data. With our cross-tissue analysis, which scanned genome-wide for SBCs, we add this unique dual-tissue perspective, focused on obesity-driven, adipose-origin, NAFLD, to the body of previous NAFLD studies.^2^^,^^9^ Although previous work has assessed the connection between either NAFLD and the adipose transcriptome^54^ or NAFLD and the liver transcriptome,^55^ our study made use of RNA-seq data measured from both adipose and liver in the same individuals.

This dual-tissue RNA-seq data first allowed us to conduct a comparative WGCNA analysis to discover adipose-liver communication within a single set of individuals. Molecular crosstalk has been detected previously using a similar method,^56^ but never on human adipose-liver interaction. Our cross-tissue design was also crucial in identifying our SBCs, because we were able to remove the liver NAFLD DE genes from the adipose DE genes to pinpoint genes specifically involved in the adipose origin of NAFLD. Detecting adipose aware SBCs is important, because adipose tissue is known to secrete a wide array of signalling proteins,^57^ which opens up the possibility for capturing the specific adipokines associated with NAFLD in serum. Additionally, our cross-tissue approach adds value to our MR method, because our IVs were derived from the list of adipose aware DE genes, which required transcriptomic data from both tissues. This allowed us to robustly demonstrate a possible unidirectional pathway, from *cis* regulatory variants of adipose aware DE genes, to elevated serum TGs, to increased NAFLD risk. In a previous paper, Yuan et al. observed a positive effect of TGs on NAFLD with MR analysis, using genetic SNPs as IVs in a NAFLD cohort of 8400+ cases and 770,000+ controls,^58^ but our study also ties the origin of the MR signal to the subcutaneous adipose tissue by utilizing cross-tissue data to obtain IV SNPs from adipose aware DE genes.

Our selection criteria for SBCs, which are specifically tailored to a cross-tissue transcriptomic design, provide additional value to our study. The HPA secretome has been applied previously to search for biomarkers,^59^ and the GTEx median TPM data have been applied to study NAFLD^60^; however, we applied the two resources in combination as a set of filtering criteria for SBCs. These publicly available datasets allowed us to implement a crucial element of our study design, i.e. the selection of the adipose aware NAFLD DE genes encoding secreted proteins which are likely to be detectable in serum. Thus, our SBCs are DE in adipose tissue but not in the liver for the three key NAFLD traits, steatosis, fibrosis and NASH, and in addition are highly expressed in adipose tissue, secreted to serum, and expressed substantially more in the adipose tissue than in the liver. In addition, the observed significant correlations between the adipose expression of SBCs and NAFLD-associated liver networks also support the possibility that the protein products of the SBC genes are markers of the liver NAFLD status in serum.

Due to our integrative filtering design, *CCDC80* and *SOD3* are likely to be indicators of NAFLD. Both of these genes meet all of our filtering criteria for SBCs, and our significant genome-wide permutation results show that they explain more variance in fibrosis and NASH than all other pairs of genes. Although our knockdown results do not prove that *CCDC80* and *SOD3* are causal in the pathogenesis of NAFLD, a biomarker can be effective whether or not it is causal. Our differentiation experiments suggesting that *SOD3* and *CCDC80* modulate genes involved in adipogenesis demonstrate their premise as indicators of the onset of NAFLD, either as responsive or causal players, and provide support for our computational methods of discovery. Additionally, previous work on the function of both genes aligns with our results.^61^, ^62^, ^63^, ^64^

As we observed in our study, *CCDC8*0 has been shown to associate positively with adverse metabolic traits, including fatty liver.^61^^,^^63^ We observed significantly increased differential expression of *CCDC80* in individuals with NAFLD, using 3 independent tests comparing individuals with steatosis, fibrosis, and NASH to those with healthy livers. In line with our human results, in a previous mouse model, *CCDC80* knockdown decreased plasma TGs.^61^ In a human study, *CCDC80* was quantified in serum, and serum *CCDC80* levels correlated positively with obesity risk, inflammation markers, and liver steatosis.^63^ It has been proposed that *CCDC80* increases hypertriglyceridemia by decreasing the expression of *LPL*, a key catalyst in hydrolysis of TGs.^61^ Gong et al. observed that *CCDC80* knockdown in vascular smooth muscle cells *in vitro* increased *LPL* expression, while *CCDC80* overexpression decreased *LPL*.^61^ In agreement with this evidence, we observed an upregulation of *LPL* in our *CCDC80* knockdown in preadipocytes during adipogenesis at day 7. Additionally, we observed that the key transcription factor of fatty acid biosynthesis, *SREBF1*, was significantly upregulated at day 7 in the *CCDC80* knockdown during adipogenesis. *SREBF1* is widely accepted as a transcription factor promoting adipogenesis.^12^ *SREBF1* exhibits a steep and sustained increase in expression during the induction stage of adipogenesis,^12^ preceding the increases in expression of other known master adipogenesis regulators *PPARG*^65^ and *CEBPA*.^12^^,^^66^ Thus, our results suggest that *CCDC80* may contribute to the pathogenesis of NAFLD by preventing adipose tissue from performing its vital functions through adipogenesis.

When treating human liver HepG2 cells with *CCDC80* recombinant protein, we observed that the *CCDC80* recombinant protein significantly modulated the expression of 9 genes, several of which have previously been linked to NAFLD and related liver pathologies, including *PPARA*, *NFE2L2*, *MOGAT1*, and *ZNF638*.^14^^,^^15^^,^^67^, ^68^, ^69^, ^70^ *PPARA* is a transcription factor that is considered a master regulator of fatty acid metabolism in the liver,^14^ and has been negatively associated with NAFLD in multiple previous studies.^15^^,^^67^ *NFE2L2* activation has previously been shown to protect against liver steatosis, fibrosis, and NASH in obese mice.^68^ In line with these previous findings and our own adipose tissue *CCDC80* NAFLD DE and *CCDC80* knockdown results, we observed a significant downregulation of *PPARA*, *NFE2L2*, *MOGAT1*, and *ZNF638* in the *CCDC80* treated liver HepG2 cells when compared to the control cells. Overall, these cellular results strengthen our converging findings, pinpointing *CCDC80* as a potential new SBC for NAFLD.

Our results also corroborate evidence supporting *SOD3* as an adipose origin biomarker for NAFLD. We observed significantly decreased differential expression of *SOD3* in individuals with NAFLD, using 3 independent tests comparing individuals with steatosis, fibrosis, and NASH to those with healthy livers. *SOD3* is seen as a protective factor against oxidative stress, which has been shown to be a major contributor to the pathogenesis of NAFLD. *SOD3* knockdown in human adipocytes caused increased accumulation of TGs,^64^^,^^71^ and global *SOD3* knockout mice exhibited increased obesity and insulin resistance.^64^ Adipocyte diameters in the white adipose tissue of mice overexpressing *SOD3* on a high-fat diet were significantly smaller than those of control mice on a high-fat diet, and were almost identical to control mice on a regular chow diet.^72^ Previous work suggests that *SOD3* functions as a protective mechanism against NAFLD development by inhibiting the expression of inflammatory genes in adipose tissue,^72^ which aligns with our hypothesis that NAFLD onset is triggered by dysfunctional and inflamed adipose tissue. Gao et al. also detected *SOD3* protein in the supernatant of human adipocytes, suggesting it is secreted by the adipose tissue.^64^ Additionally, we observed that *LEP* was significantly downregulated in the *SOD3* knockdown at day 4 during adipogenesis. *LEP* encodes an adipokine, leptin, which is secreted from adipose tissue that acts on the brain, playing a major role in energy homeostasis and satiety signalling.^73^ It has been shown that *LEP* acts as the primary link between adipose tissue and the brain, in a negative feedback loop that decreases hunger urges with increasing energy intake and fat accumulation.^73^ However, this same system has been demonstrated to break down in obesity, where *LEP* deficiency and/or *LEP* resistance hinder the ability of the body to balance energy intake and expenditure.^73^ Taken together, our *SOD3* knockdown results suggest that *SOD3* may protect against NAFLD by promoting effective energy homeostasis.

We observed that the *SOD3* recombinant protein significantly altered the expression of 2 genes in liver HepG2 cells, most importantly *RNF128*. *RNF128* is a transcription factor, the overexpression of which has previously been shown to enhance hepatic lipid accumulation and increase the expression of lipid metabolic genes in mice and liver cells,^16^ and *RNF128* knockout mice were also found to be resistant to developing liver steatosis on a high-fat diet compared to control mice, indicating that its absence plays a protective role against NAFLD.^16^ In line with these previous findings and our own adipose tissue *SOD3* NAFLD DE and *SOD3* knockdown results, we observed a significant downregulation of *RNF128* in the *SOD3* treated liver HepG2 cells when compared to the control cells. These results further support our existing findings, and highlight *SOD3* in addition to *CCDC80* as a potential new SBC for NAFLD.

Although an effective serum biomarker is not necessarily causal, our MR results suggest that some genetic regulators of adipose aware DE genes, notably of the SBC *VEGFB*, may play an important role in NAFLD. Our MR analysis used a set of 6 IVs, all of which regulate local adipose aware DE gene expression and colocalise with TG GWAS signals, to show that elevated serum TGs have a possible unidirectional effect on increasing NAFLD risk. We also show that there are zero such IVs to support the reverse causal hypothesis that serum TG levels are impacted by the NAFLD status. This result provides converging evidence for our adipose-origin NAFLD hypothesis, and places our adipose aware DE genes at the beginning of the pathway connecting obesity-induced adipose dysfunction to NAFLD. Additionally, we observed that the *VEGFB cis*-eQTL rs2845885 produces a significant result when used as the sole IV in MR analysis. This result is especially important, because it implies that *VEGFB*, which already satisfies all of our SBC filtering criteria, may be critical in the progression of adipose-origin NAFLD. On top of this, our regression analyses adding the *VEGFB* adipose expression to models only utilizing serum TGs as an explanatory variable for NAFLD imply that *VEGFB* expression captures components of the NAFLD variance that are not captured by TGs alone. This makes *VEGFB* both a promising biomarker candidate, and a potential target for therapeutic interventions for NAFLD. *VEGFB* is significantly downregulated in our NASH DE results, which aligns with our *cis*-eQTL and MR results, as well as previous studies.^74^ The SNP rs2845885 is negatively associated with *VEGFB* expression, but positively associated with serum TGs. Previous studies in mice found that *VEGFB* KO resulted in increased fat accumulation,^74^ while *VEGFB* transduction suppressed adipose inflammation.^75^ Taken together, these results suggest that *VEGFB* possibly functions as an inhibitor of NAFLD by promoting the normal physiological functions of adipose tissue.

*CCDC80*, *SOD3*, and *VEGFB*, along with the full list of 10 SBCs, should be considered for inclusion in future serum biomarker panels to diagnose NAFLD. Six of the 10 SBCs have been previously measured in serum, using ELISA kits and other related methods.^63^^,^^64^^,^^76^, ^77^, ^78^, ^79^ Additionally, new results from another group studying the UK Biobank^80^ support the assertion that the three SBCs *CCDC80*, *CD3*00LG, and *TIMP3* are secreted to serum and can be measured in serum, and that their protein levels may indicate NAFLD. Sun et al. found that the serum levels of these three proteins correlated significantly with BMI in a cohort of more than 54,000 UK Biobank participants (p < 1.000 × 10^−651^, 1.585 × 10^−30^, and 2.512 × 10^−119^, respectively). BMI can be used as a proxy for obesity, and thus these previous results indicate that a significant proportion (30%) of the SBCs are detectable in serum, are correlated with BMI, and may correlate with obesity-related NAFLD. The other seven SBCs were not measured in this previous study,^80^ and the authors did not test any NAFLD traits. Thus, further investigation specifically in NAFLD cases and controls is still needed. Also, although our study leveraged the list of secreted proteins from the HPA, the majority of our analysis was done in a transcriptomic paradigm, under the assumption that mRNA abundance of the SBCs in adipose tissue is an effective proxy for their corresponding protein levels in serum. Future vetting of our SBCs should involve comparing their protein levels in serum between individuals with and without NAFLD.

One of the limitations of our study is that our discovery cohort consists of individuals with morbid obesity originating from a relatively genetically homogeneous European population, the Finns.^81^ It is crucial to follow up our work with future studies in populations underrepresented in genomics, including Indigenous, Latin American, African, and Southeast Asian populations. Here, we made use of the European KOBS cohort because it was the first to make our dual-tissue NAFLD study design possible. Thus, even though the previous mouse studies (described above) and our experimental adipogenesis knockdown studies and MR results support the role of the identified adipose aware NAFLD DE genes in obesity-related NAFLD, it would be important to further investigate these findings in other European and more diverse human multi-tissue transcriptomics cohorts with liver histology available for study when those become available. Additionally, the participants of the liver snRNA-seq cohort were older than the participants of the adipose snRNA-seq cohort, which may affect the identification of the adipose and liver cell-type marker genes due to unknown age effects. Future investigation is needed to determine whether this age difference could be a confounding factor in the identification of cell-type marker genes from snRNA-seq data.

Currently, NAFLD can be diagnosed using liver biopsy, which necessitates an invasive surgery or inpatient procedure, or abdominal imaging (MRI, magnetic resonance spectroscopy (MRS), or elastography), which is costly and time consuming.^2^ Furthermore, NAFLD often remains undiagnosed and is therefore grossly underdiagnosed,^2^ emphasizing the pressing need for SBCs. We envision that our cell culture-validated SBCs have strong potential to be developed into an effective blood panel for NAFLD, which could be used in the primary care setting as initial screening before the current invasive and expensive diagnostic techniques. This could allow for more efficient primary care screenings for NAFLD, including the clinically grave form of NAFLD, fibrosis, and ultimately improve patient health by catching and treating NAFLD earlier in its development. We also envision that high-risk patients for NAFLD, specifically obese patients, could greatly benefit from this diagnostic option.

In conclusion, leveraging paired dual-tissue RNA-seq data from the same obese individuals’ adipose tissue and liver along with liver histology-based NAFLD diagnosis, we discover a set of genes whose adipose expression is likely to contribute to NAFLD via obesity-induced adipose dysfunction. We also selected 10 serum biomarker candidates for NAFLD from this set. We identified *CCDC80* and *SOD3*, which explain maximum variance in fibrosis and NASH compared to all SBCs, as the key SBCs, and followed up this conclusion with functional knockdown experiments throughout adipogenesis. We also discovered proof-of-principle genetic evidence for the involvement of adipose aware DE genes, especially the SBC, *VEGFB*, in a possible unidirectional pathway from adipose dysfunction to NAFLD via serum TGs. Our methodology can be extended to study cross-tissue links and discover SBCs in any complex disease, provided that a cohort of RNA-seq data from multiple tissues in the same individuals is available, along with genotypes and detailed phenotype data. Overall, identifying genes and SBCs involved in tissue-tissue crosstalk using our integrative transcriptomics pipeline could contribute to improved understanding and earlier clinical detection and diagnosis of complex diseases in the future.

## Contributors

N. D., M. A., and P. P. designed the study. N. D., M. A., S. H. T. L., D. Z. P., Z. M., P. P., and J. S. S. performed or supervised the computational and statistical analyses. U. T. A., T. Ö., and M. U. K. conducted the siRNA knockdown experiments in SGBS preadipocytes and collected the adipogenesis RNA-seq samples. M. W. provided human preadipocytes for the preadipocyte differentiation experiment. I. S. and M. U. K. conducted the HepG2 recombinant protein experiments and collected the RNA-seq samples. J. P., D. K., M. A., and P. P. collected the KOBS RNA-seq data. D. K., P. P., and J. P. collected the KOBS genotype data. V. M. and J. P. were responsible for KOBS clinical and histological data. M. A., J. N. B, P. P., and J. R. P. generated the liver snRNA-seq data. K. H. P., M. A., and P. P. generated the adipose snRNA-seq data. M. L. collected the METSIM genotype data. S. S. D. contributed to data visualization. N. D. and P. P. wrote the manuscript, and all other authors read, edited, and approved of the manuscript. N. D., M. A., and P. P. directly accessed and verified the underlying data reported in the manuscript.

## Data sharing statement

Access to the existing KOBS bulk RNA-seq data^17^^,^^82^ and the Finnish Twin and CRYO adipose snRNA-seq data^17^^,^^22^^,^^83^ are described in the original publications for each cohort. Access to the METSIM genotype data is available from NIH dbGaP under accession number phs000919.v1.p1. The GTEx median TPM data, for all tissues including subcutaneous adipose tissue and liver, is available from the GTEx portal under the label “Median gene-level TPM by tissue” (https://gtexportal.org/home/datasets). The Human Protein Atlas (HPA) secretome data is available from the HPA website under the label “Blood Protein”. UK Biobank data are available for bona fide researchers through the application process (https://www.ukbiobank.ac.uk/learn-more-about-uk-biobank/contact-us). The liver snRNA-seq data^31^ are available from NIH GEO under accession number GSE189175. All code used for computational analysis is available at https://github.com/ndarci/adipose-dysfunction-nafld.

## Declaration of interests

J. N. B. received consulting fees from GLG, and support for attending meetings and/or travel from the American Gastroenterology Association (AGA), at least once during the last 36 months. The other authors declare that they have no competing interests.

## Appendix A Supplementary data

Supplementary Figs. S1–S4 and Tables S10 and S14–S17

Caption for supplementary material
