# Supplementary material for: Cross-tissue omics analysis discovers ten adipose genes encoding secreted proteins in obesity-related non-alcoholic fatty liver disease
Source: eBioMedicine. 2023 May 22;92:104620. doi: 10.1016/j.ebiom.2023.104620 (PMC10277924; doi:10.1016/j.ebiom.2023.104620)
Supplement: Supplementary Figs. S1–S4 and Tables S10 and S14–S17 [file mmc13.docx]

Supplementary Information: Cross-tissue omics analysis discovers ten adipose genes encoding secreted proteins in obesity-related non-alcoholic fatty liver disease

Nicholas Darci-Maher^1^, Marcus Alvarez^1^, Uma Thanigai Arasu^2^, Ilakya Selvarajan^2^, Seung Hyuk T. Lee^1^, David Z. Pan^1^, Zong Miao^1^, Sankha Subhra Das^1^, Dorota Kaminska^1,3,4^, Tiit Örd^2^, Jihane N. Benhammou^5^, Martin Wabitsch^6^, Joseph R. Pisegna^7^, Ville Männistö^8^, Kirsi H. Pietiläinen^9,10^, Markku Laakso^11^, Janet S. Sinsheimer^1,12,13^, Minna U. Kaikkonen^2^, Jussi Pihlajamäki^3,14^, Päivi Pajukanta^1,15,16*^

^1^Department of Human Genetics, David Geffen School of Medicine at UCLA, Los Angeles, USA.

^2^A. I. Virtanen Institute for Molecular Sciences, University of Eastern Finland, Kuopio, Finland.

^3^Institute of Public Health and Clinical Nutrition, University of Eastern Finland, Kuopio, Finland.

^4^Division of Cardiology, David Geffen School of Medicine at UCLA, Los Angeles, USA.

^5^Vatche and Tamar Manoukian Division of Digestive Diseases and Gastroenterology, Hepatology and Parenteral Nutrition, David Geffen School of Medicine at UCLA and VA Greater Los Angeles HCS, Los Angeles, USA.

^6^Division of Pediatric Endocrinology and Diabetes, Department of Pediatrics and Adolescent Medicine, University of Ulm, Ulm, Germany

^7^Department of Medicine and Human Genetics, Division of Gastroenterology, Hepatology and Parenteral Nutrition, David Geffen School of Medicine at UCLA and VA Greater Los Angeles HCS, Los Angeles, USA.

^8^Department of Medicine, University of Eastern Finland and Kuopio University Hospital, Kuopio, Finland.

^9^Obesity Research Unit, Research Program for Clinical and Molecular Metabolism, Faculty of Medicine, University of Helsinki, Helsinki, Finland

^10^Obesity Center, Abdominal Center, Helsinki University Hospital and University of Helsinki, Helsinki, Finland

^11^Institute of Clinical Medicine, Kuopio University Hospital, University of Eastern Finland, Kuopio, Finland.

^12^Department of Biostatistics, UCLA Fielding School of Public Health, Los Angeles, USA.

^13^Department of Computational Medicine, David Geffen School of Medicine at UCLA, Los Angeles, USA.

^14^Department of Medicine, Endocrinology and Clinical Nutrition, Kuopio University Hospital, Kuopio, Finland.

^15^Bioinformatics Interdepartmental Program, UCLA, Los Angeles, USA.

^16^Institute for Precision Health, David Geffen School of Medicine at UCLA, Los Angeles, USA.

*Correspondence: Päivi Pajukanta, MD, PhD

Professor of Human Genetics

David Geffen School of Medicine at UCLA

University of California, Los Angeles (UCLA)

Gonda Center, Room 6357B, 695 Charles E. Young Drive South

Los Angeles, California 90095-7088, USA

Email: ppajukanta@mednet.ucla.edu

Supplementary Figures

#
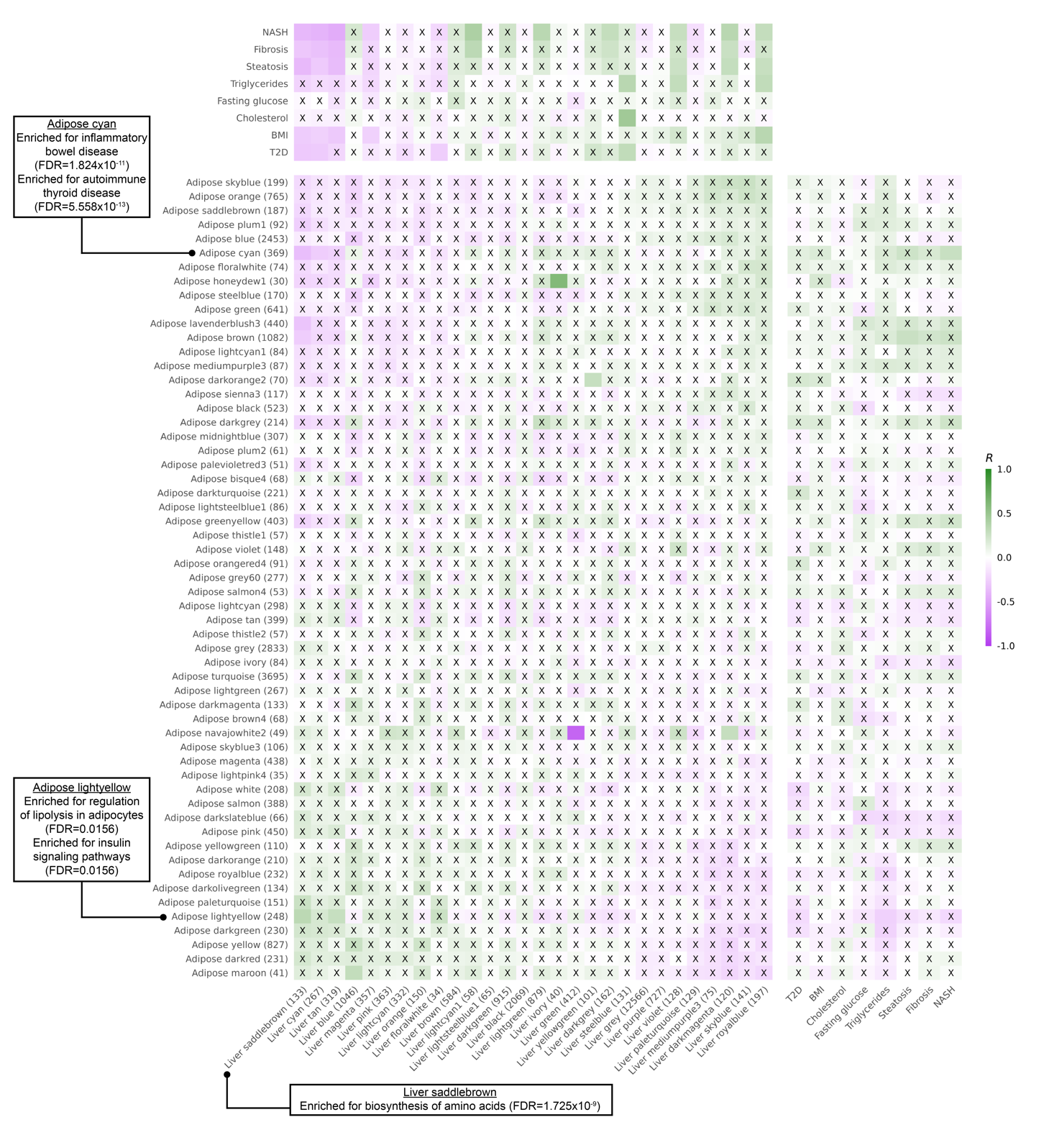


**Supplementary Figure 1: Co-expression networks that are associated with functional pathways and related to non-alcoholic fatty liver disease (NAFLD) correlate across an individual’s subcutaneous adipose and liver tissue.** We ran Weighted Gene Co-expression Network Analysis (WGCNA) on the KOBS adipose and liver gene RNA-seq expression data, generating two independent gene co-expression networks representing adipose and liver gene expression in the same individuals (see Methods). To calculate module-module and module-phenotype correlations, we summarized the expression of all genes in a module using the module eigengene, defined as the first principal component (PC) of expression. In all plots, green boxes represent positive correlation, while purple boxes represent negative correlation, as indicated by the legend. Bottom left panel: pairwise Pearson correlations between all modules in the adipose and liver networks. X-axis represents the names of modules in the liver co-expression network, where the number in parenthesis is the number of genes in the network. Y-axis represents the names of modules in the adipose co-expression network. “X” indicates non-significance after Bonferroni correction (adjusted p>0.05), with the number of tests equal to the product of the number of modules in the adipose and liver co-expression networks. Adipose and liver modules are ordered by the first PC of correlation coefficients. Upper left panel: pairwise Pearson correlations between all modules in the liver network and selected NAFLD and obesity-related phenotypes in the KOBS cohort. X-axis represents modules in the liver co-expression network, and Y-axis represents phenotypes (T2D = type 2 diabetes; BMI = body mass index; NASH = non-alcoholic steatohepatitis). “X” indicates non-significance after Bonferroni correction, with the number of tests equal to the product of the number of modules in the liver co-expression network and the number of phenotypes tested. Bottom right panel: pairwise Pearson correlations between all modules in the adipose network and the same phenotypes. X-axis represents the phenotypes, and Y-axis represents the modules in the adipose co-expression network. “X” indicates non-significance after Bonferroni correction, with the number of tests equal to the product of the number of modules in the adipose co-expression network and the number of phenotypes tested. Black boxes below and to the left of the plot indicate functional enrichment results from WebGestalt for the key cross-tissue correlated networks.


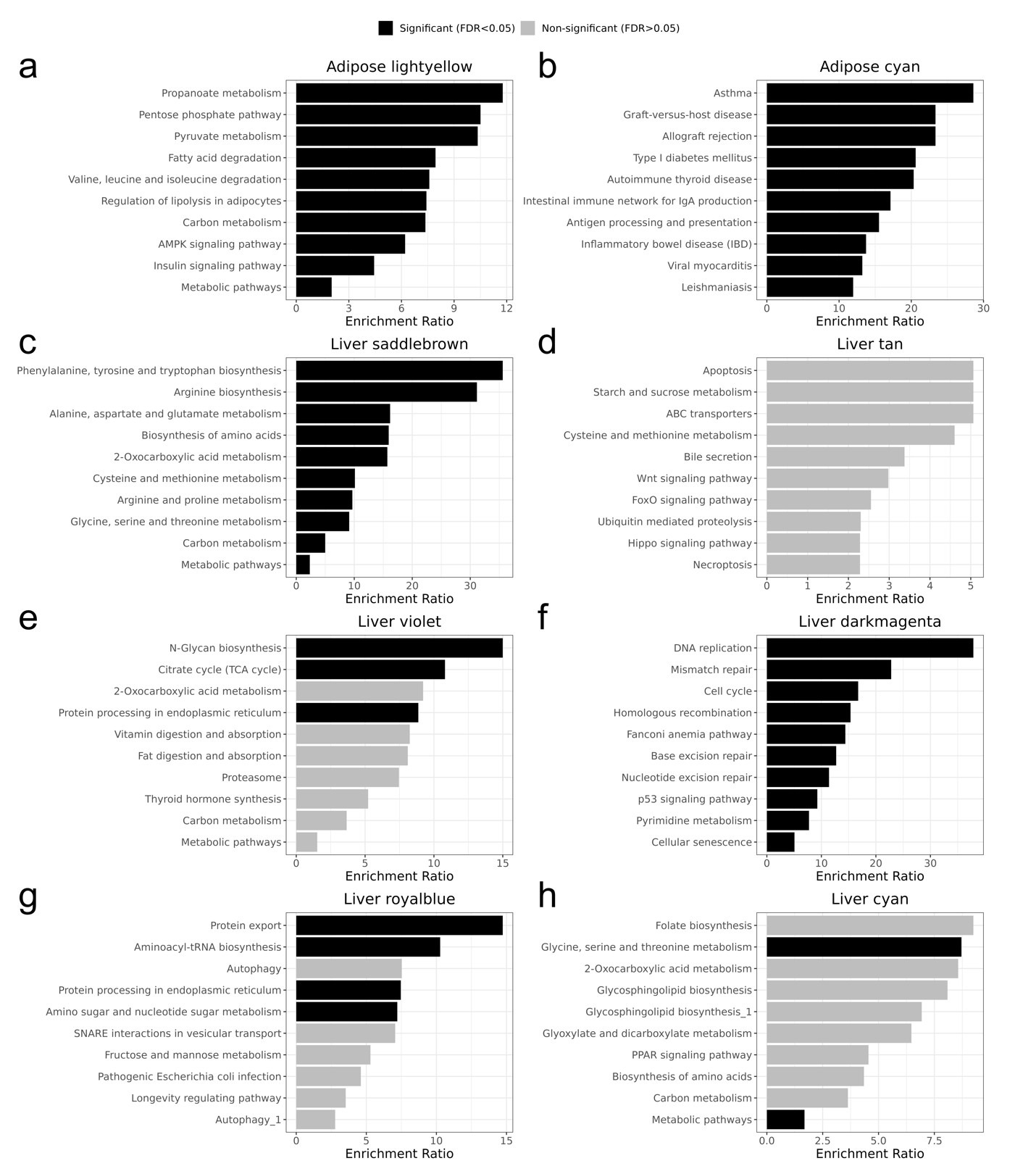


**Supplementary Figure 2: Key adipose and liver co-expression networks are enriched for cellular pathways related to adipogenesis and NAFLD.** We ran pathway enrichment analysis using WebGestalt on the 8 key co-expression networks, including the adipose and liver networks that correlated across tissues, as well as the liver networks that correlated with adipose expression of the serum biomarker candidate genes (SBCs). In all plots, X-axis represents the enrichment ratio as calculated by WebGestalt, and Y-axis represents the name of the enriched pathway. Plot title indicates the adipose or liver co-expression network that was tested for enrichment. Black bars represent pathways with significant enrichment (FDR<0.05) among the network genes, and grey bars represent non-significant enrichment.

#
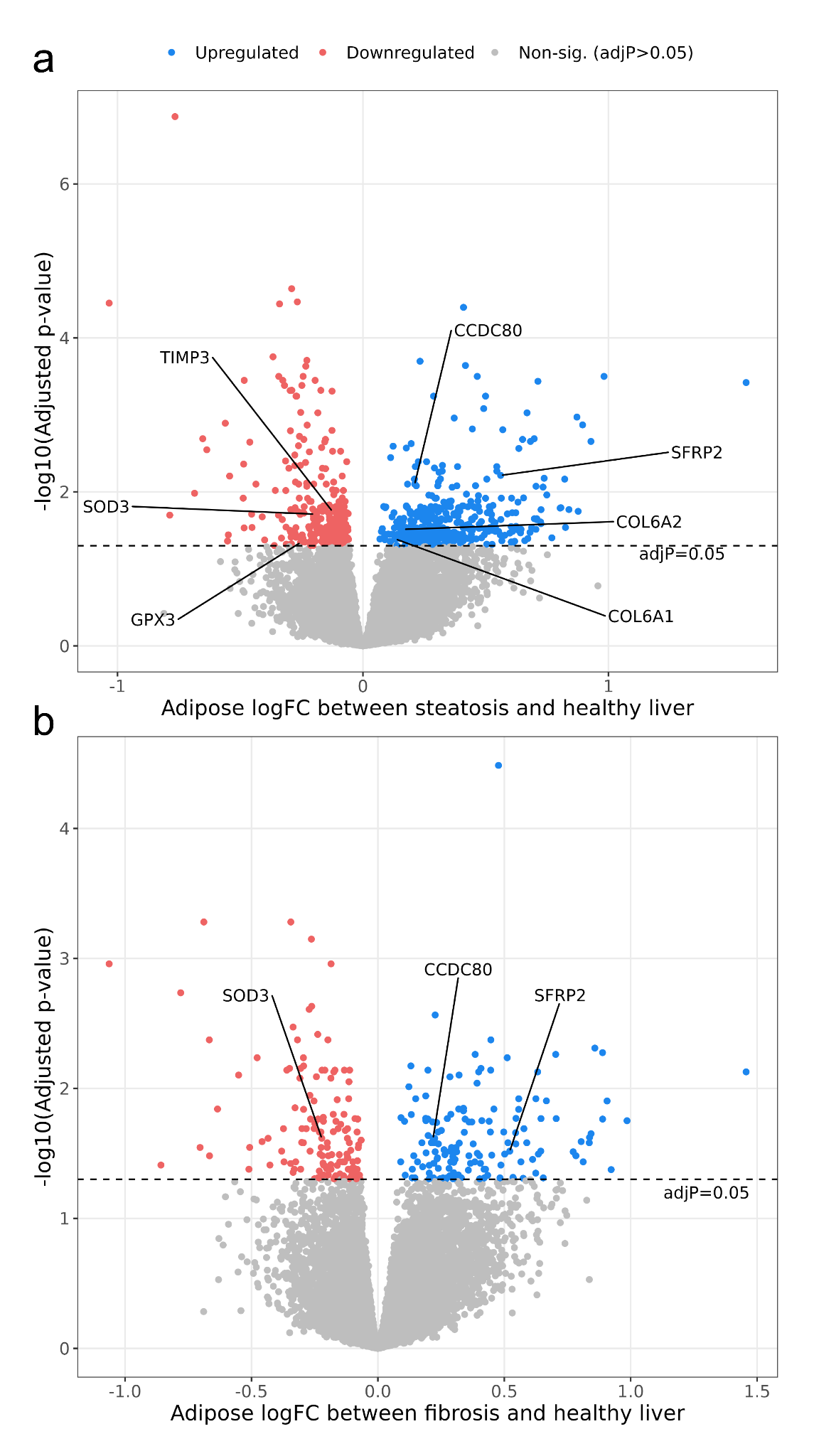


**Supplementary Figure 3: A total of 680 genes are differentially expressed (DE) for steatosis, and 273 genes for fibrosis, in the subcutaneous adipose tissue from the KOBS participants with morbid obesity.** We performed DE analysis on bulk RNA-seq data from subcutaneous adipose biopsies in the KOBS cohort, comparing individuals with the non-alcoholic fatty liver disease (NAFLD) traits diagnosed by liver histology to those with healthy livers (n=237 for steatosis; n=199 for fibrosis). Gene counts represent numbers of genes DE for NAFLD in the subcutaneous adipose tissue before filtering for serum biomarker candidates (SBCs). Of the 953 adipose DE genes, 680, 273, and 663 genes are DE for steatosis, fibrosis, and non-alcoholic steatohepatitis (NASH) (see Figure 2), respectively. Volcano plots show the results of the steatosis (n=237) (a) and fibrosis (n=199) (b) DE analyses in the adipose tissue. In both plots, the X-axis represents log fold-change (logFC) in adipose bulk RNA-seq data from individuals with the particular NAFLD trait and those with healthy livers. The Y-axis represents the negative log of the DE p-value, adjusted for multiple testing with the Benjamini-Hochberg procedure. Significant SBCs identified in our filtering steps are highlighted.


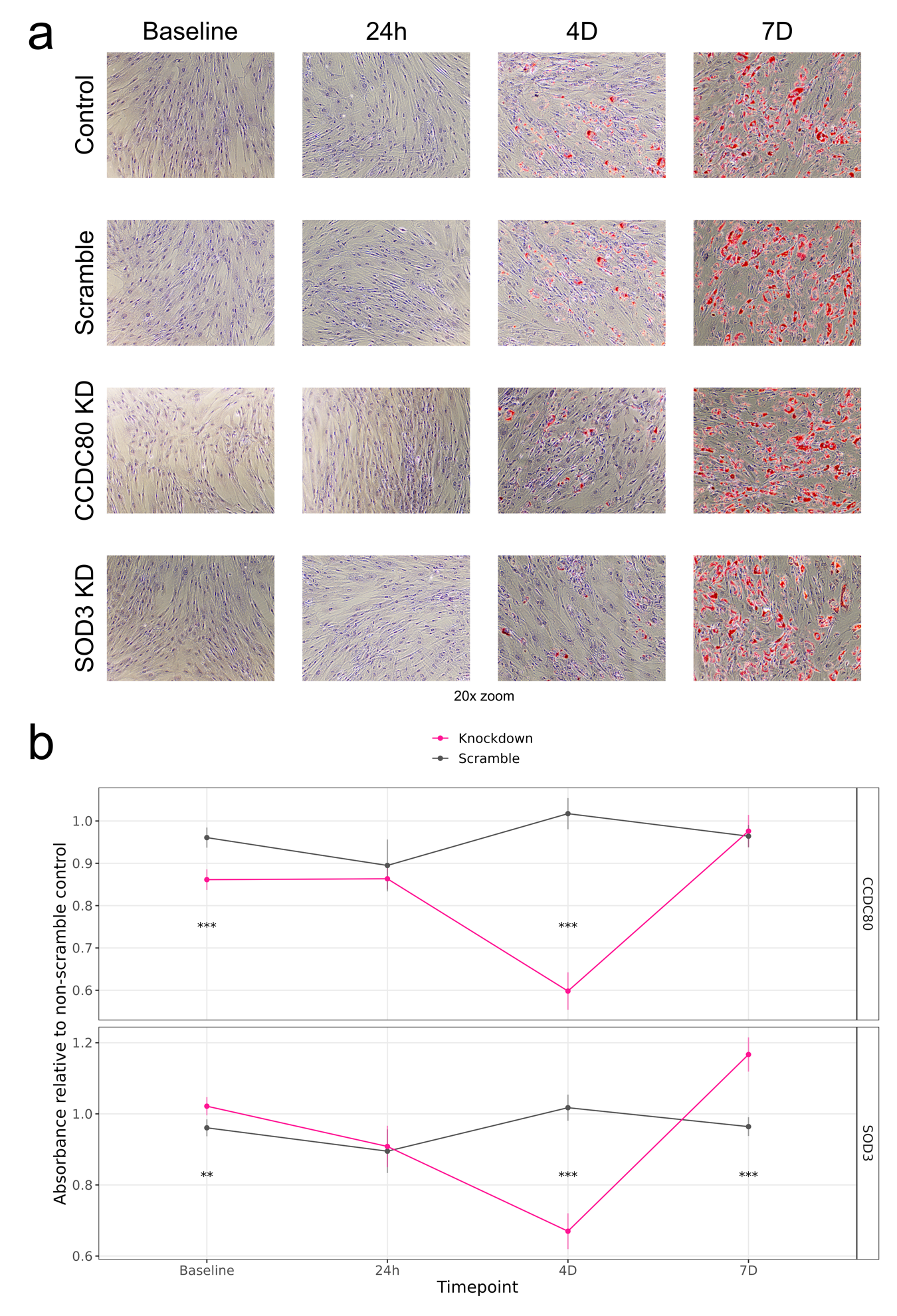


**Supplementary Figure 4: Lipid staining with Oil Red O (ORO) demonstrates that *CCDC80* and *SOD3* knockdown delay the accumulation of lipids in SGBS preadipocytes as they differentiate to preadipocytes.** We knocked down *CCDC80* and *SOD3* in separate cultures of human preadipocyte cells during adipogenesis (see Methods), and observed the lipid accumulation at the same time points where gene expression was measured. We then quantified the intensity of the ORO staining by measuring the absorbance of 492nm wavelength light, and normalized the intensity to the cell number. (a) Cell images taken with the EVOS Core XL microscope at 20x zoom. Rows indicate each of the four experimental conditions: non-transfected control cells, scrambled siRNA control, *CCDC80* knockdown (KD), and *SOD3* KD. Columns indicate time points of adipogenesis: baseline, 24 hours, 4 days, and 7 days. Red colour is ORO staining. (b) Mean ORO intensity in the *CCDC80* and *SOD3* knockdown compared to the mean intensity in the scrambled control. The X-axis represents the time point of the adipocyte differentiation. The Y-axis represents the absorbance of 492nm wavelength light, normalized to the cell number and relative to the intensity of the non-scrambled control at each time point. Error bars represent the mean absorbance ± one standard deviation. The colours represent the experimental condition (knockdown or scrambled control). The facets represent the *CCDC80* and *SOD3* knockdowns. Each condition-timepoint combination within each facet represents n=4 samples, and the total experiment included n=48 samples. Annotations indicate the significance of a t-test between the knockdown and scramble samples at a given timepoint: “***” = p<0.001; “**” = p<0.01; “*” = p<0.05.

| **Supplementary Table 10: Model selection statistics from the best subsets analysis for steatosis, fibrosis, and NASH in the KOBS adipose expression data.** | | | | | |
| --- | --- | --- | --- | --- | --- |
| **Phenotype** | **Genes in model** | **r^2^** | **Adj. r^2^** | **BIC** | **Best subset** |
| Steatosis | COL6A2 | 0.055 | 0.051 | -2.437 | * |
| Steatosis | COL6A2+GPX3 | 0.075 | 0.067 | -1.974 |  |
| Steatosis | COL6A1+COL6A2+GPX3 | 0.075 | 0.063 | 3.471 |  |
| Fibrosis | SOD3 | 0.061 | 0.056 | -1.983 |  |
| Fibrosis | CCDC80+SOD3 | 0.102 | 0.093 | -5.502 | * |
| Fibrosis | CCDC80+SOD3+SFRP2 | 0.111 | 0.098 | -2.332 |  |
| NASH | CCDC80 | 0.108 | 0.102 | -8.707 |  |
| NASH | CCDC80+SOD3 | 0.166 | 0.156 | -14.813 | * |
| NASH | CCDC80+TIMP3+SOD3 | 0.181 | 0.166 | -12.685 |  |
| NASH | CCDC80+TIMP3+SOD3+MGP | 0.195 | 0.175 | -10.465 |  |
| NASH | CCDC80+TIMP3+SOD3+MGP+CD300LG | 0.196 | 0.171 | -5.562 |  |
| NASH | CCDC80+TIMP3+SOD3+MGP+SFRP2+CD300LG | 0.197 | 0.167 | -0.609 |  |
| NASH | CCDC80+TIMP3+SOD3+MGP+SFRP2+CD300LG+VEGFB | 0.198 | 0.162 | 4.315 |  |
| Phenotype indicates the NAFLD phenotype being tested in the best subsets analysis. Genes in model indicate the genes in the linear model represented by each row. r^2^ indicates r-squared statistic representing the proportion of variance in the phenotype explained by the genes in the model (plus covariates). Adj. r^2^ indicates adjusted r-squared statistic taking number of variables into account. BIC stands for Bayesian Information Criterion, used to choose the best subset of genes. Best subset denotes the best subset chosen for each phenotype (based on minimum BIC) with an asterisk. | | | | | |

| **Supplementary Table 14: Correlation analysis between adipose expression of serum biomarker candidate genes (SBCs) and liver tissue co-expression network module eigengenes from Weighted Gene Co-expression Network Analysis (WGCNA). Only correlations passing Bonferroni corrected adj. p-value<0.05 are shown.** | | | | | |
| --- | --- | --- | --- | --- | --- |
| **SBC** | **Liver network** | **r** | **r^2^** | **P-value** | **Adj. p-value** |
| COL6A1 | tan | -0.228 | 0.052 | 4.49E-04 | 4.49E-02 |
| GPX3 | violet | -0.267 | 0.071 | 3.76E-05 | 3.76E-03 |
| SFRP2 | darkmagenta | 0.229 | 0.052 | 4.36E-04 | 4.36E-02 |
| SFRP2 | violet | 0.268 | 0.072 | 3.46E-05 | 3.46E-03 |
| TIMP3 | royalblue | -0.294 | 0.086 | 5.15E-06 | 5.15E-04 |
| TIMP3 | tan | 0.243 | 0.059 | 1.85E-04 | 1.85E-02 |
| TIMP3 | cyan | 0.283 | 0.08 | 1.16E-05 | 1.16E-03 |
| TIMP3 | saddlebrown | 0.345 | 0.119 | 6.30E-08 | 6.30E-06 |
| SBC indicates the serum biomarker candidate, the adipose expression of which correlates with the key liver networks. Liver network indicates the liver WGCNA network that correlates with SBC adipose expression. r indicates Pearson's r correlation statistic between the adipose expression of the SBC and the liver network module eigengene. r^2^ indicates the squared r statistic. P-value indicates the p-value for significance of the correlation between the SBC adipose expression and the liver network module eigengene. Adj. p-value indicates the p-value adjusted for multiple testing using Bonferroni correction. | | | | | |

| **Supplementary Table 15: Logistic regression statistics showing the increased proportion of variance explained in steatosis, fibrosis, and NASH by the addition of VEGFB expression to serum triglyceride measurements alone.** | | | | |
| --- | --- | --- | --- | --- |
| **Phenotype** | **Variables in model** | **Model type** | **Pseudo-r^2^** | **AUC** |
| Steatosis | Triglycerides | Logistic | 0.141 | 0.683 |
| Steatosis | VEGFB+Triglycerides | Logistic | 0.152 | 0.693 |
| Fibrosis | Triglycerides | Logistic | 0.17 | 0.699 |
| Fibrosis | VEGFB+Triglycerides | Logistic | 0.19 | 0.718 |
| NASH | Triglycerides | Logistic | 0.234 | 0.737 |
| NASH | VEGFB+Triglycerides | Logistic | 0.278 | 0.766 |
| Phenotype indicates the NAFLD phenotype being tested in the logistic regression analysis. Variables in model indicates the variables in the logistic regression model represented by each row. Model type indicates the type of regression model used in the model represented by each row. Pseudo-r^2^ indicates the Nagelkerke pseudo-r-squared statistic approximating the proportion of variance in the phenotype explained by the variables in the model (plus covariates). AUC indicates the area under the receiver operating characteristic (ROC) curve for the model represented by each row. | | | | |

| **Supplementary Table 16: Linear regression statistics showing the increased proportion of variance explained in steatosis, fibrosis, and NASH by the addition of VEGFB expression to serum triglyceride measurements alone.** | | | | |
| --- | --- | --- | --- | --- |
| **Phenotype** | **Variables in model** | **Model type** | **r^2^** | **Adj. r^2^** |
| Steatosis | Triglycerides | Linear | 0.104 | 0.075 |
| Steatosis | VEGFB+Triglycerides | Linear | 0.112 | 0.079 |
| Fibrosis | Triglycerides | Linear | 0.127 | 0.094 |
| Fibrosis | VEGFB+Triglycerides | Linear | 0.142 | 0.104 |
| NASH | Triglycerides | Linear | 0.175 | 0.136 |
| NASH | VEGFB+Triglycerides | Linear | 0.209 | 0.166 |
| Phenotype indicates the NAFLD phenotype being tested in the linear regression analysis. Variables in model indicates the variables in the linear regression model represented by each row. Model type indicates the type of regression model used in the model represented by each row. r2 indicates the r-squared statistic representing the proportion of variance in the phenotype explained by the variables in the model (plus covariates). Adj. r^2^ indicates the adjusted r-squared statistic taking the number of variables into account. | | | | |

| **Supplementary Table 17: Elastic net regression statistics showing the increased proportion of variance explained in steatosis, fibrosis, and NASH by the addition of VEGFB expression to serum triglyceride measurements alone.** | | | | | |
| --- | --- | --- | --- | --- | --- |
| **Phenotype** | **Variables in model** | **Model type** | **r^2^** | **VEGFB coeff.** | **TG coeff.** |
| Steatosis | Triglycerides | Elastic net | 0.048 | NA | 2.203 |
| Steatosis | VEGFB+Triglycerides | Elastic net | 0.054 | 0 | 1.855 |
| Fibrosis | Triglycerides | Elastic net | 0.041 | NA | 1.049 |
| Fibrosis | VEGFB+Triglycerides | Elastic net | 0.085 | -0.521 | 2.054 |
| NASH | Triglycerides | Elastic net | 0.114 | NA | 1.322 |
| NASH | VEGFB+Triglycerides | Elastic net | 0.181 | -0.694 | 2.498 |
| Phenotype indicates the NAFLD phenotype being tested in the elastic net regression analysis. Variables in model indicates the variables in the elastic net regression model represented by each row. Model type indicates the type of regression model used in the model represented by each row. r^2^ indicates the r-squared statistic representing the proportion of variance in the phenotype explained by the variables in the model (plus covariates). VEGFB coeff. indicates the coefficient of the VEGFB expression in the elastic net model ("NA" represents a model without VEGFB as an explanatory variable). TG coeff. indicates the coefficient of triglycerides (TG) in the elastic net model. | | | | | |
